# Advances in neuroprostheses: interfaces, materials, and applications

**DOI:** 10.1186/s40580-026-00552-2

**Published:** 2026-05-26

**Authors:** Enhui He, Kangming Chen, Shishuo Liu, Haisong Chen, Yu Xiao, Rongrong Chen, Peiqin Tu, Gang Pan, Peng Lin

**Affiliations:** 1 The State Key Laboratory of Brain-Machine Intelligence, College of Computer Science and Technology, Zhejiang University, Hangzhou, China; 2 Nanhu Brain-Computer Interface Institute, Hangzhou, China; 3 MOE Frontier Science Center for Brain Science and Brain-Machine Integration, Zhejiang University, Hangzhou, China

**Keywords:** Neuroprostheses, Brain–computer interface, Neuromorphic, Neural electrodes, Sensing materials

## Abstract

Neuroprostheses have become a pivotal technology for restoring sensory, motor, and cognitive functions, offering transformative therapeutic strategies for neurological disorders by bridging or bypassing damaged neural pathways through electronic systems. However, achieving long-term stability and high-fidelity interaction between biological and electronic systems remains a significant challenge due to the mismatch at the neural interface. This review examines the critical role of nanotechnology in building high performance neuroprostheses across six key classes: motor, visual, tactile, language, memory and olfactory. A system architecture of the neuroprostheses is proposed that highlights two critical interfaces, namely, “neural-electronic” and “environment-electronic” interfaces. We survey recent advances in materials and devices that shape better neural electrodes and novel sensors, and discuss the potential utilization of neuromorphic computing for efficient edge processing in neuroprostheses. This review aims to outline future trajectories toward high-throughput bidirectional interaction, biomimetic encoding, and adaptive closed-loop systems, aspiring to achieve seamless integration between electronic systems and biological neural circuitry.

**Graphical abstract:**

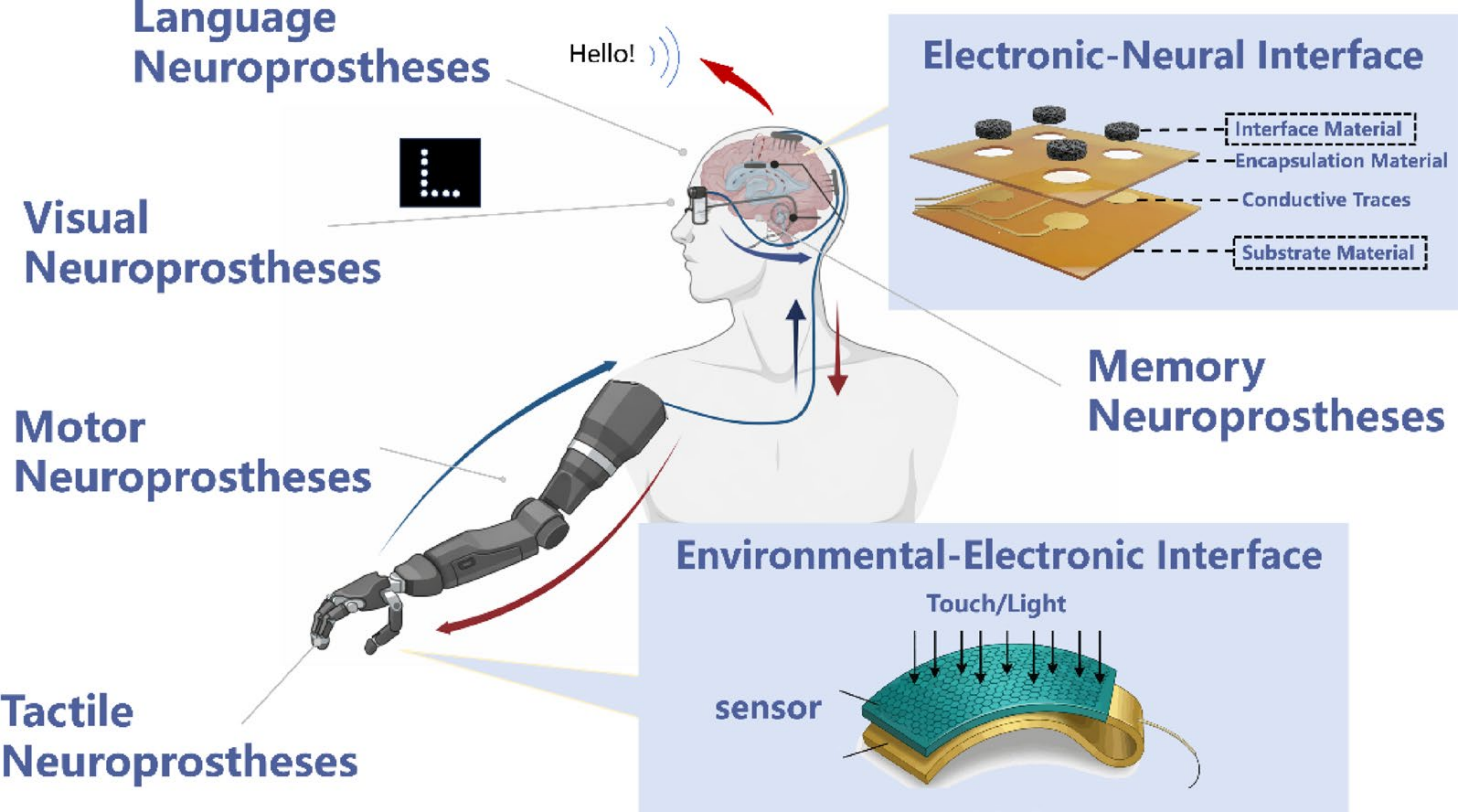

## Introduction

The human nervous system is a highly integrated, closed-loop network that processes information through hierarchical and bidirectional signaling across sensory receptors, neurons, synapses, and neural circuits. Distinct neural functions, such as vision, motor control, and cognition, are formed by specific neural pathways in different brain regions. These pathways are closely interfaced with the external environment through bidirectional interactions. Exogenous stimuli is transduced into electrophysiological signals by specialized receptors, transmitted via afferent pathways, and integrated by the brain into perceptual and cognitive processes. Reciprocally, the brain generates descending efferent commands that control motor execution, speech production, and other functional outputs. This precise functional segregation offers high processing capability and brings vulnerability, since each pathway is unique and damage to specific regions that cannot be compensated by others. Consequently, neurological disorders, ranging from physical trauma and stroke to neurodegenerative conditions such as Alzheimer, often result in the irreversible disruption of neural pathways. Conventional therapies like drug intervention and surgical procedures often fail to restore lost functions [1]. Fortunately, neuroprostheses have emerged as a transformative solution, offering functional recovery by bypassing damaged neural circuits involved in sensing, transmission or actuation [2, 3]. By directly interfacing with the biological circuitry, neuroprostheses re-establish the lost dialogue within the fragmented neural networks to recover sensory, motor, and cognitive capabilities that would otherwise remain permanently compromised.

Over the past few decades, the neuroprostheses have made significant progress. For example, visual neuroprostheses can elicit light perception (e.g. phosphenes) of coarse shapes and object motions in blind patients through electrical stimulation, and contribute to functional vision restoration that enables blind patients to independently perform daily tasks such as crossing streets [4]. Similarly, motor neuroprostheses allow paralyzed patients to control robotic limbs by decoding their motor imagery (MI) [5], while speech neuroprostheses assist patients to convert their speaking intentions into computer-generated speech output [6]. Despite these remarkable advancements, current neuroprostheses still face significant functional limitations that hinder their widespread clinical adoption and long-term efficacy. In most cases, the interactions between the prostheses and the nervous systems remain insufficient to replicate natural perception.

A primary challenge lies in reliable and high fidelity biological and electronic interactions. Biological tissues and electronic materials are inherently different. Implanting many electrodes into the brain is beneficial for high bandwidth communications, but can also lead to extended chronic inflammatory responses and glial scarring. Although innovations in electrode materials have improved performance in both neural signal recording and stimulation [7], their long-term biocompatibility remains a key challenge. Deficiencies in electrode performance cast limits on spatial resolution and restricting their ability to capture complex, high-dimensional neural patterns. In the meantime, patients with perception losses require artificial sensory to feed information to the brain, which demands an array of sensors, as well as efficient and capable processing modules to convert external signals into brain-decodable stimulation. Advances in sensing materials enable neuroprostheses to acquire external sensory signals with high efficiency and sensitivity [8], and the use of artificial intelligence plays an increasing role in designing signal decoders and encoders in neuroprostheses. However, power consumption and thermal constraints pose significant barriers to integrating high performance processing hardware into implantable systems. The lack of on-device edge computing significantly restricts the closed-loop operation, constraining the adaptive and dynamical reconfigurability needed to respond to real-time neural feedback or environmental changes. Consequently, while neuroprostheses have demonstrated functional recovery in specific scenarios, they remain far from achieving the high-fidelity and long-term stability required for seamless functional restoration.

This article presents a systematic review of neuroprostheses, covering both applications and systems designs. While there have been relevant reviews on neuroprostheses focusing on specific applications (such as visual [9, 10] and motor prostheses [11]), key technologies (such as closed-loop control [12], large-scale neural signals decoding [13], and neural interfaces [14, 15]), or emerging directions (such as personalized neuroprosthetics [16], regenerative electrodes [17], and neuromorphic hardware [18]), there is a lack of reviews addressing system-level designs to generalize key functional modules across different neuroprostheses. In this review, we aim to provide an abstract hardware architecture for neuroprosthetic systems, and summarize main information flows in various types of neuroprostheses. First, we introduce the major categories of neuroprostheses (motor, visual, tactile, language, memory, and olfactory), emphasizing their recent progress and representative clinical applications. Subsequently, we examine the core system modules, with particular focus on material advances at two critical interfaces: the neural-electronic interface, and the environment-electronic interface. We also highlight the need for compute-in-memory and neuromorphic computing as the core module for efficient processing in future closed-loop neuroprostheses. Finally, we discuss the common challenges faced by current neuroprostheses and outline future development directions, with the goal of providing a roadmap for the continued advancement of the field.

## Recent developments of major neuroprostheses

Currently, the primary landscapes of neuroprostheses includes motor, tactile, visual, language, memory, and olfactory. These neuroprostheses restore or enhance specific neural functions either by decoding neural activities to drive external devices or by delivering artificial sensory and cognitive information through targeted neural stimulation. Recent advances in these representative neuroprosthetic systems have demonstrated broad therapeutic potential in restoring sensory, motor, and cognitive functions. However, the performance and clinical application of current neuroprostheses remain constrained by several fundamental limitations. Therefore, a comprehensive review of the present applications and development of major neuroprostheses is essential not only to illustrate the current status of the field, but also to identify key technological bottlenecks.

### Motor neuroprostheses

**Fig. 1:**
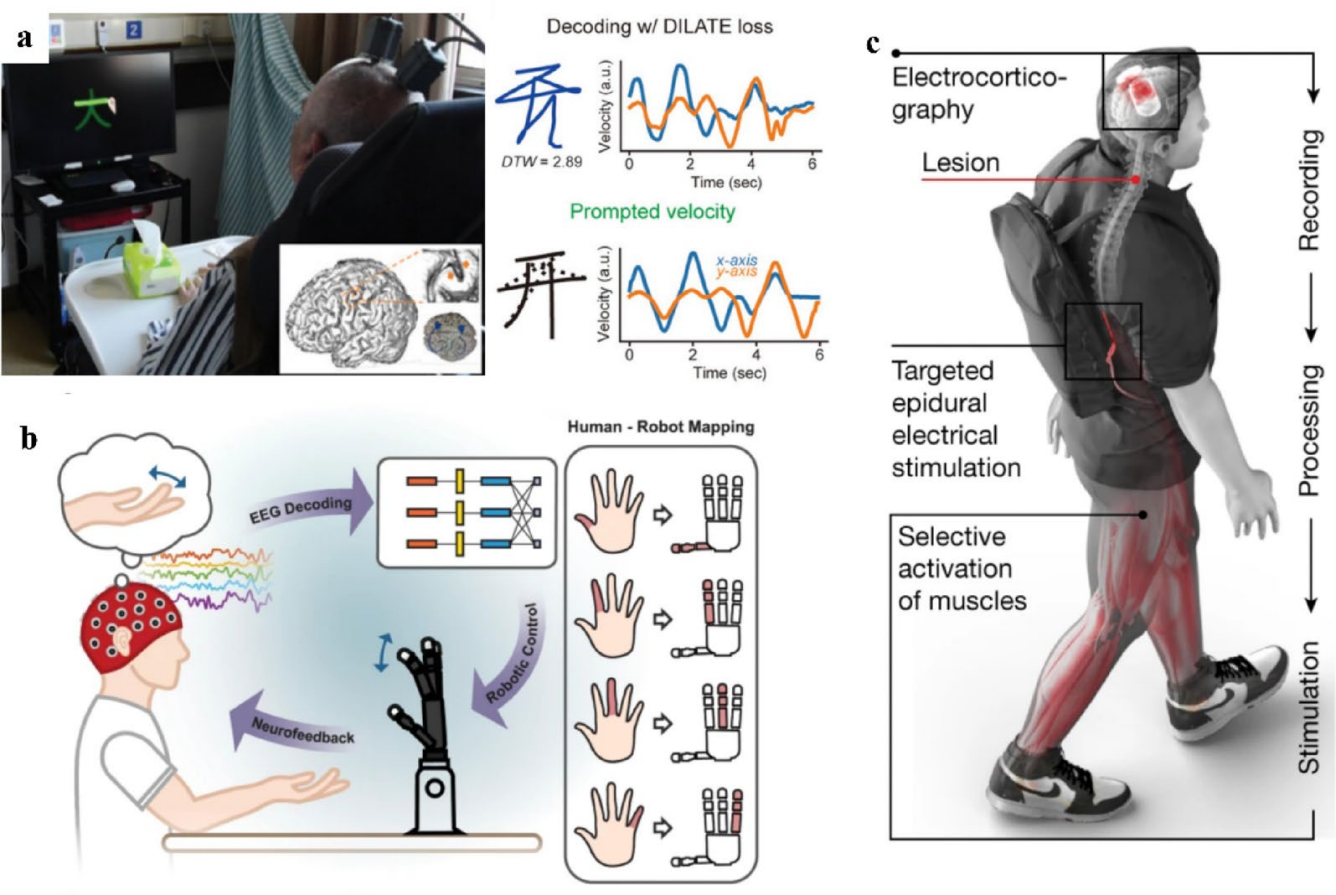
Motor neuroprostheses. **a** Invasive motor prostheses that reconstruct handwriting trajectory based on motor cortex, reprint with permission from Ref [5]. **b** Non-invasive motor prostheses that control a robotic hand using motor imagery, reprint with permission from Ref [24]. **c** Brain-spine-based prostheses for motor restoration, reprint with permission from Ref [25]

Motor neuroprostheses restore motor function through two primary paradigms: (1) decoding movement intentions to control external assistive devices, such as robotic arms, and (2) using electrical stimulation to activate downstream neural pathways which restore voluntary limb movement. Both approaches have demonstrated effective performance and growing clinical potential. Fundamentally, these systems are designed to decode neural activities, particularly from the motor cortex, into actuator control signals. Early animal studies established feasibility ranging from decoding movement intentions in rats to real-time robotic arm control in non-human primates [19, 20]. Subsequent clinical studies have further validated these capabilities. For instance, Hochberg et al. showed that individuals with tetraplegia could achieve two-dimensional control of a neural cursor and robotic arm [21]. Subsequently, they enabled continuous three-dimensional control for complex daily tasks such as reaching, grasping and drinking [22], while further increasing dexterity to support fine hand movements [23]. More recently, Xu et al. used invasive electrodes to enable a participant to control a robot arm for handwritten Chinese characters with 91.1% accuracy across 1,000 characters (Fig. 1a) [5], highlighting the potential of motor neuroprostheses for complex behaviors.

Despite these successes, the invasive technologies still face significant challenges, including surgical risks, long-term signal stability, and high costs. Consequently, non-invasive brain–computer interfaces (BCIs) have attracted growing interest due to their safety, portability and affordability. Among these, electroencephalography (EEG)-based systems have recently demonstrated substantial application potential, owing to advances in signal processing and artificial intelligence that have significantly improved decoding performance of EEG signals, effectively mitigating their traditional limitations such as low signal-to-noise ratio (SNR) and poor spatial resolution. For instance, Forenzo et al. showed that motor imagery (MI) could support two-dimensional robotic arm control, enabling participants to complete continuous reach–grasp–transport–place tasks at an average of seven successful cup-transport trials within 5 min [26]. Ding et al. further combined motor execution (ME) and MI to achieve finer robotic finger control, achieving classification accuracy of 80.56% for two-finger tasks and 60.61% for three-finger tasks. These results demonstrated the feasibility of independent finger control using non-invasive methods (Fig. 1b) [24].

In addition to controlling external devices, motor neuroprostheses can restore function by directly activating paralyzed limbs through electrical stimulation guided by decoding brain signals. For instance, Lorach et al. combined cortical decoding with spinal stimulation, using recorded neural signals from implanted cortical electrodes to produce real‑time stimulation delivered to the lumbosacral cord. This closed‑loop scheme not only supported independent ambulation but also promoted reorganization of impaired pathways, suggesting a shift from purely assistive interfaces toward interventions that drive neurorehabilitation (Fig. 1c) [25].

### Visual neuroprostheses

**Fig. 2:**
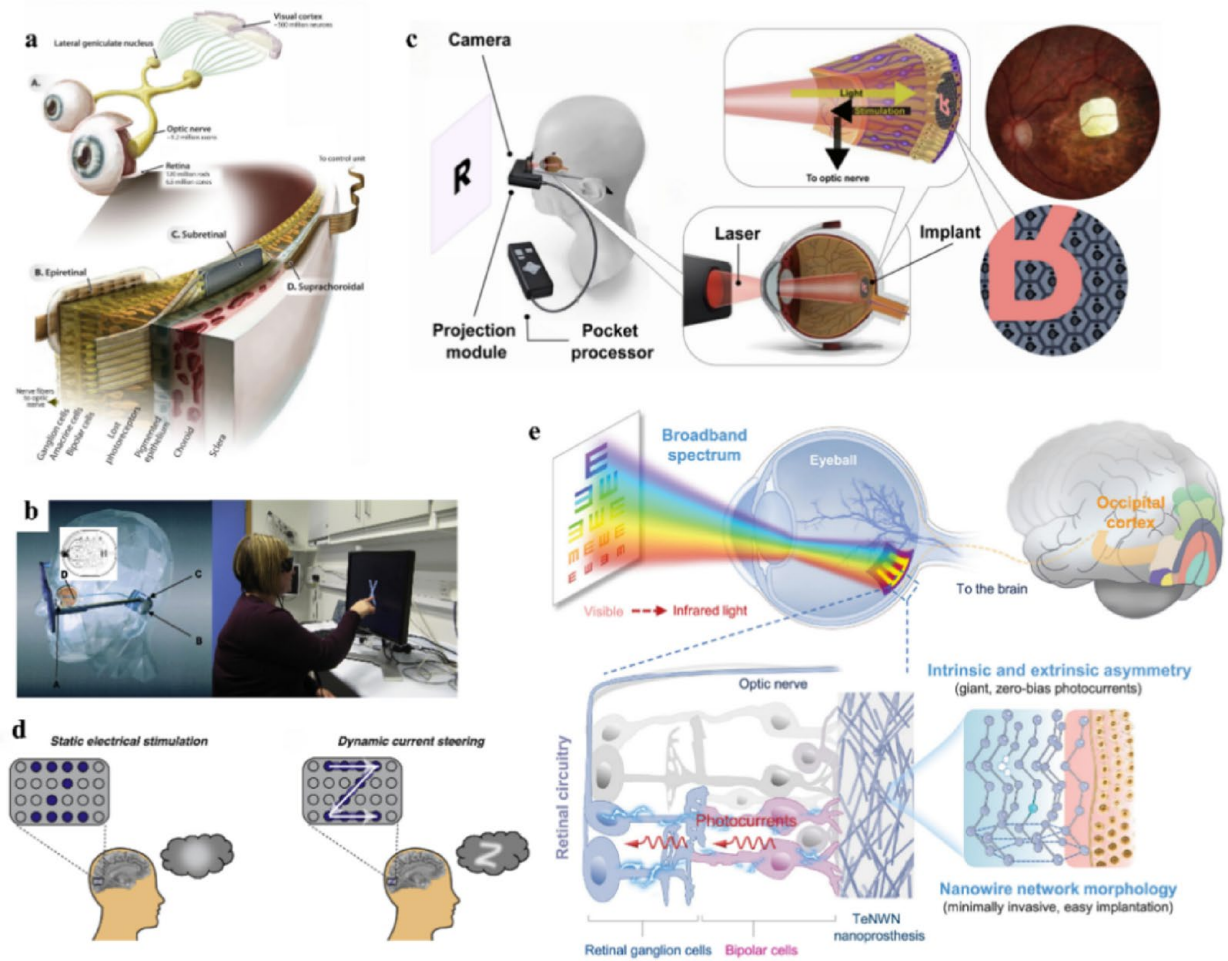
Visual neuroprostheses. **a** A schematic overview of visual neuroprostheses according to implantation sites, including epiretinal, subretinal, suprachoroidal, and cortical prostheses, reprint with permission from Ref [27]. **b** The Argus system which participants rely on to recognize the letter ‘x’, reprint with permission from Ref [28]. **c** The PRIMA retinal prosthesis system with wireless photovoltaic architecture for subretinal stimulation, reprint with permission from Ref [29]. **d** Comparison of static electrical stimulation (left) and dynamic current steering (right) strategies in the visual cortex, highlighting differences in elicited perceptual patterns, reprint with permission from Ref [30]. **e** The schematic of vision restoration via a next-generation TeNWN-based nanoprosthesis, enabling mice to perceive infrared light, reprint with permission from Ref [31]

Visual neuroprostheses aim to restore visual function in patients suffering from retinal degeneration or neurodegenerative diseases. By bypassing damaged components of the visual pathway, these devices convert visual information into electrical signals that directly stimulate the remaining intact neural pathways, thereby eliciting visual phosphenes in the brain. Visual neuroprostheses are broadly classified into retinal prostheses and cortical prostheses (Fig. 2a) [27].

#### Retinal visual prostheses

Retinal visual prostheses are designed for patients with severe retinal degeneration, including retinitis pigmentosa (RP) and age-related macular degeneration (AMD). Based on the implantation sites, these devices are classified as epiretinal, subretinal, and suprachoroidal prostheses. Epiretinal prostheses, which are the earliest to reach clinical trials, are placed on the inner retinal surface, primarily stimulate ganglion cells. Subretinal prostheses, implanted beneath the photoreceptor layer of the retina, preserve more intrinsic retinal processing and therefore produce more naturalistic visual phosphenes. Suprachoroidal prostheses, placed between the sclera and choroid, offer greater surgical safety and lower invasiveness with a reduced spatial resolution.

The Argus II is a representative epiretinal prosthesis with 60 electrodes, achieving a best grating acuity of 20/1262, and supporting object localization, motion discrimination, and simple characters recognition (Fig. 2b) [28, 32–34]. The PRIMA is a wireless photovoltaic subretinal prosthesis with 378 near-infrared light sensitive pixels that can stimulate bipolar cells to restore light phosphenes. Recent trials reported marked functional improvement with some participants reaching 20/42 acuity using optical zoom and recovering a certain degree of reading ability (Fig. 2c) [29, 35]. Regarding suprachoroidal prostheses, a 44-channel BVT system showed stable performance for up to 2.7 years and enabled independent use in daily life [36].

Recent developments in retinal prostheses have primarily followed two converging directions. The first focuses on improving resolution and visual field by increasing stimulation density and coverage range. For example, Laura et al. developed an epiretinal prosthesis with 2,215 stimulation sites which cover 46.3° visual field [37], while Zhou et al. reported a full-field hydrogel–polymer epiretinal array expandable to 34 mm in diameter, corresponding to an visual field of 113° [38]. The second direction aims to reduce invasiveness and enhances long-term safety through alternative stimulation modalities. Leong et al. developed a flexible photoacoustic retinal prosthesis that converted pulsed near-infrared light into localized ultrasound, achieving 51 μm lateral resolution with a temperature rise below 1 °C [39]. Jiang et al. further proposed a flexible ultrasound-driven retinal prosthesis enabling wireless acoustic-to-electrical conversion and reducing the thermal burden associated with conventional electrical stimulation [40]. Overall, these advances indicate that retinal prostheses are evolving toward high-density, wide-field, wireless, flexible, and multi-modal platforms.

#### Cortical visual prostheses

Cortical visual prostheses are implanted in the visual cortex and bypass the eye, retina, and optic nerve. Therefore, compared with retinal prostheses, they may be applicable to most forms of acquired blindness except those resulting from cortical injury.

Several cortical prosthetic systems have demonstrated effective vision restoration capability. The CORTIVIS project combined a 100-channel Utah electrode array with a custom ASIC and successfully elicited phosphenes in all tested subjects [41, 42]. Similarly, the Orion system, based on a 60-electrode cortical array, enabled blind participants to localize fist-sized targets and detect moving bar [43]. However, phosphene elicitation alone remains far from reconstructing the rich spatiotemporal structure of natural vision.

Current research in cortical visual prostheses has converged on two major directions: increasing stimulation channels to improve spatial resolution, and refining stimulation protocols to generate more structured and naturalistic phosphenes. In the first direction, Chen et al. implanted a 1024-channel Utah electrode array into the visual cortex of monkeys and applied a sequential stimulation strategy to generate phosphenes of motion direction with classification accuracy of 70–80% [44]. In terms of stimulation strategies, Fernández et al. implanted a Utah electrode array at the visual cortex, and used static multi-electrode stimulation patterns to enable participant to identify simple letters such as “I”, “L”, and “C” [45]. Concurrently, Daniel et al. used fast activation sequence in electrodes with optimized parameters to elicit light-point trajectories in the visual field. Clinical results showed that participants could accurately discriminate complex shapes such as “Z” and “W” (Fig. 2d) [30]. Moreover, the use of new materials has enabled visual capabilities beyond the visible spectrum. For instance, visual prostheses based on tellurium nanowire networks have enabled mice to perceive infrared light (Fig. 2e) [31]. Furthermore, recent work has demonstrated that visual prostheses can not only be used for visual function reconstruction, but also promote visual recovery, which is significant for the treatment of visual impairments [46].

### Tactile neuroprostheses

**Fig. 3:**
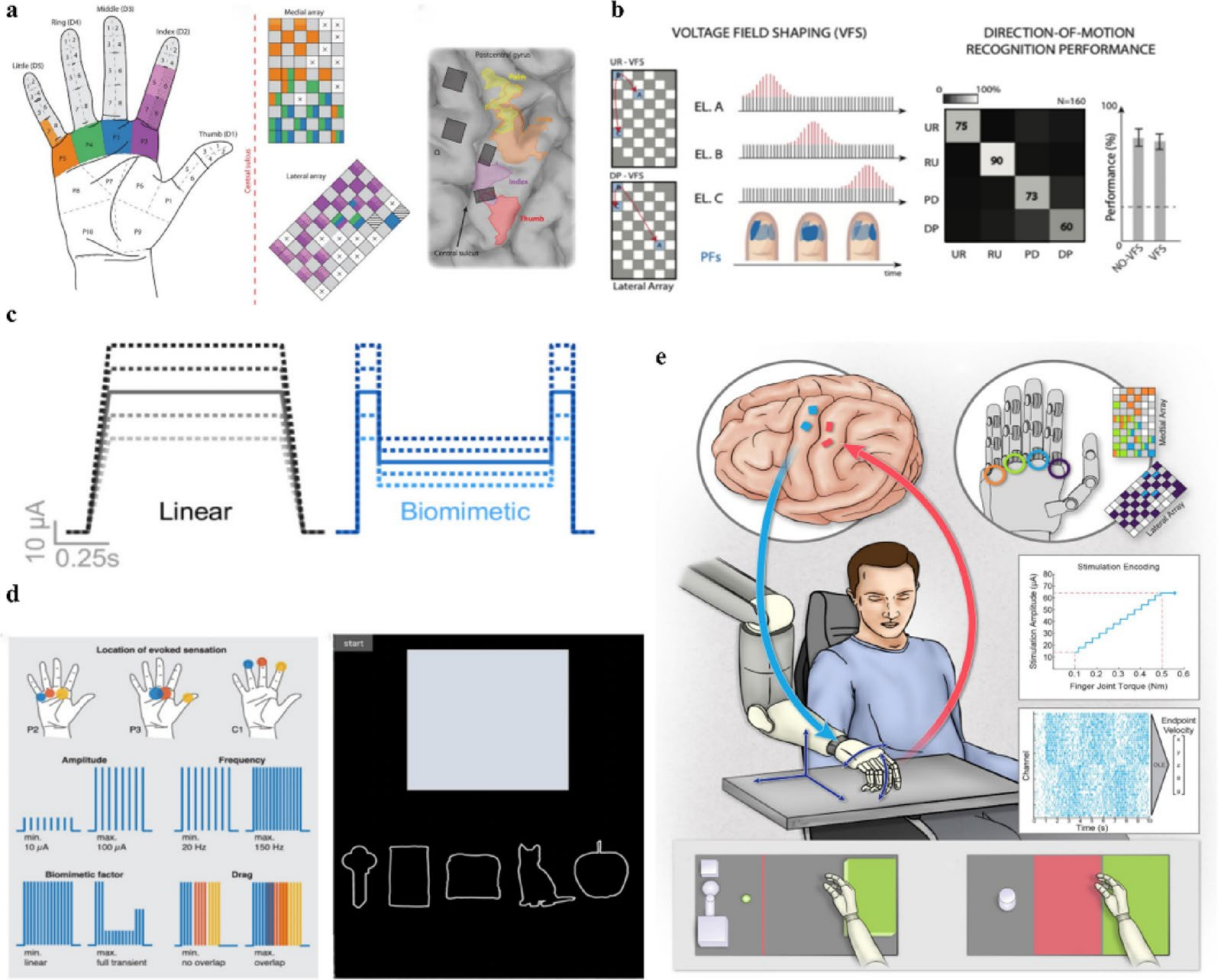
Tactile neuroprostheses. **a** The spatial correspondence between microelectrode array stimulation sites and evoked palmar sensory locations, reprint with permission from Ref [48]. **b** The fingertip perception of motion direction via dynamic stimulation (left) and recognition accuracy (right), reprint with permission from Ref [50]. **c** The comparison of traditional linear stimulation encoding patterns (left) and biomimetic encoding patterns (right), reprint with permission from Ref [51]. **d** Stimulation-evoked sensory locations and stimulation parameters (left); Object property perception, including compliance, temperature, and friction (right), reprint with permission from Ref [52]. **e** The schematic of a closed-loop system integrating motor and tactile neuroprostheses, reprint with permission from Ref [53]

Tactile neuroprostheses restore somatosensory function by delivering artificial feedback on modalities such as pressure, texture, and temperature. This restoration relies on a topographic correspondence between specific brain regions and the sensory areas to which feedback is directed (Fig. 3a) [47]. Early studies focused on the linear relationship between single-parameter electrical stimulation and tactile perception. Flesher et al. found that amplitude-based electrical stimulation at different electrodes could evoke effective pressure sensations which are mapped to different limb regions [48]. However, amplitude-based encoding is limited by the just noticeable difference (JND), which constrains finer sensory discrimination. Subsequently, Callier et al. demonstrated that frequency modulation (10–200 Hz) could modulate perceptual intensity with 10–20 non-overlapping JNDs, substantially exceeding the 5–7 JNDs achieved by conventional amplitude modulation [49]. Based on these advances, tactile encoding evolved from single-parameter modulation to multi-electrode spatiotemporal patterns. For instance, Valle et al. elicited perceptions of tactile edges, apparent motion and simple shapes (e.g., T, L, C, and O) using spatially structured and sequential stimulation (Fig. 3b) [50].

Recently, biomimetic encoding has emerged as an advanced strategy for enhancing artificial tactile perception. By simulating the spike timing, frequency variation and population dynamics of natural nervous system, these approaches enable more naturalistic sensory transmission. In peripheral neural interfaces, George et al. applied a biomimetic intraneural stimulation strategy to the residual median and ulnar nerves using chronically implanted USEAs. This strategy was based on TouchSim, a computational model which simulates the population firing activities of tactile afferent nerve fibers in response to tactile stimulation of the primate fingertip. Compared with linear encoding, this population-based biomimetic strategy improved reaction speed in compliance discrimination tasks by 56% [54]. Valle et al. further developed a biomimetic encoding strategy based on the Izhikevich neuron model, which converts sensor inputs into biologically realistic spike trains by modeling neuronal firing dynamics. This strategy evoked more natural sensations delivered through implanted TIME electrodes, achieved grasp success rates above 90% in a “virtual egg test” [55]. In intracortical microstimulation (ICMS), Greenspon et al. designed dynamic biomimetic waveforms inspired by the “contact transient” response pattern of somatosensory cortical neurons which are characterized by brief high-frequency bursts at contact onset and offset with reduced activity during sustained contact [56]. Compared with linear stimulation, biomimetic encoding increased the number of discriminable levels from 8 to 11, when combined with multi-channel stimulation, it increased further to 19.5 (Fig. 3c) [51]. Similarly, Verbaarschot et al. introduced a “biomimetic factor” to regulate the amplitude ratio between sustained and transient ICMS phases, enabling participants to adjust factor in real time during testing. This approach allowed accurate discrimination of multiple tactile qualities, including warm, cool, compliant and smooth sensations (Fig. 3d) [52]. These findings support the view that biologically grounded spike generation can substantially enrich the information delivered to the nervous system.

Beyond restoring tactile perception, tactile neuroprostheses can be integrated with motor prostheses to establish closed-loop sensorimotor systems. By returning afferent feedback generated during environmental interaction, such systems improve motor accuracy, alleviate phantom limb pain, and prevent cortical deterioration associated with chronic sensory deprivation. Ultimately, this preserves plasticity within somatosensory circuits and enables more stable use of prostheses. Flesher et al. combined tactile and motor neuroprostheses to establish a complete closed-loop system, which enabled real-time control of a 5-degrees-of-freedom robotic arm with grasping via motor cortex decoding. Moreover, the addition of sensory feedback reduced grasp duration by 50% and overall task completion time by 88% (Fig. 3e) [53]. Prolonged et al. demonstrated that tactile neuroprosthesis feedback drives not only behavioral improvements but also functional recovery within central circuits, highlighting its importance for restoring motor function [57].

### Language neuroprostheses

Language neuroprostheses are designed for individuals who have lost speech capabilities due to brain injury, neurological diseases, or severe speech disorders. By decoding neural activities into intelligible text or synthesized speech, these systems aim to restore the pathway from intention to expression.

**Fig. 4:**
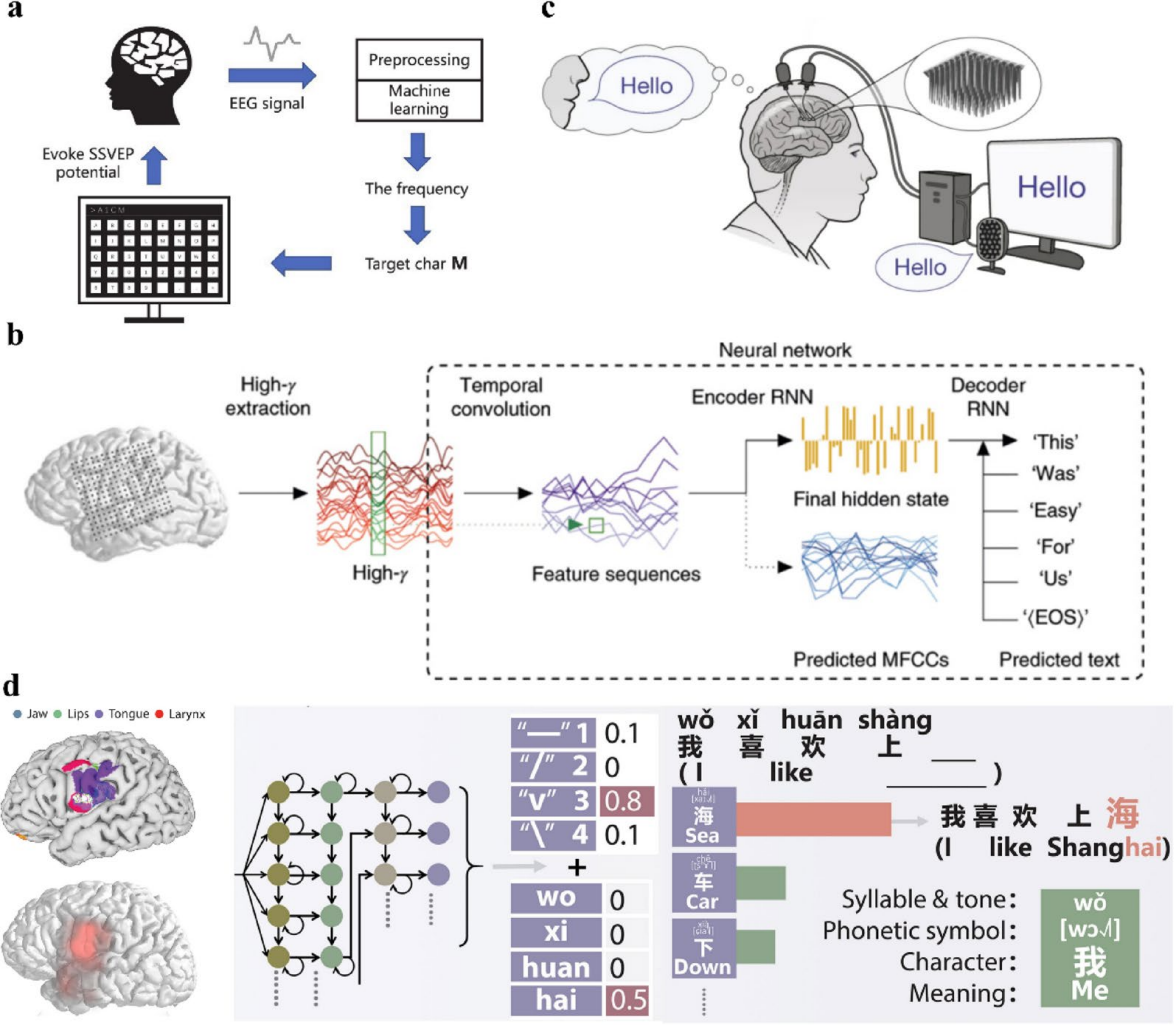
Language neuroprostheses. **a** The schematic of an SSVEP-based language neuroprostheses, reprint with permission from Ref [62]. **b** Language neuroprostheses based on ECoG electrodes, reprint with permission from Ref [63]. **c** The schematic of an intracortical microelectrode-based language neuroprosthesis, reprint with permission from Ref [64]. **d** A real-time Chinese language decoding architecture for synchronous decoding of syllables and tones, reprint with permission from Ref [65]

Non-invasive EEG dominated early work of neural speech prostheses. For individuals with preserved oculomotor control, visual P300 character matrices [58, 59] and steady-state visually evoked potential (SSVEP) systems (Fig. 4a) [60–62] have been widely adopted, enabling characters selection via fixation on flickering on-screen targets. The P300 approach relies on the brain’s event-related potential (ERP) response to attended stimuli, while SSVEP exploits neural synchronization with periodic visual inputs.

EEG-based language neuroprostheses remain constrained by limited speed and practicality, although many studies report decoding accuracy exceeding 90% [66]. Consequently, recent efforts have increasingly shifted toward invasive or semi-invasive approaches, aiming to decode language either from the motor regions of speech production or language-related cortical regions. Current mainstream approaches target motor areas, decoding speech-related motor neural activities of phonemes, letters or word generation, and enabling fluent language production. Anumanchipalli et al. developed a two-stage decoder using electrocorticography (ECoG) signals from the ventral sensorimotor cortex. They first inferred articulatory kinematic trajectories—mapping neural activities to vocal articulators’ movements—and subsequently synthesized continuous speech. The system performed effectively with as little as 25 min of training data, achieving perfect transcriptions for 43% of 25 words and phrases [67]. By contrast, Makin et al. employed an encoder–decoder architecture that directly translated ECoG sequences into English sentences. Using less than 40 min of training speech per participant (30–50 repeated readings), they achieved a mean word error rate (WER) of ~ 3% on a ~ 250-word vocabulary task (Fig. 4b) [63]. Beyond ECoG, Willett et al. used intracortical microelectrode arrays to record spiking activity in the ventral anterior motor cortex (area 6v), combining RNN-based phoneme decoding with language-model fusion to enable online text communication. Participants reached 62 words per minute with WER of 9.1% for a constrained 50-word vocabulary and 23.8% for a large 125,000-word vocabulary (Fig. 4c) [64].

However, these high-performance systems largely depend on speech-production motor regions, with decoding targets tightly coupled to orofacial articulation. In order to broaden application, particularly for aphasia populations, researchers have begun exploring direct decoding from neural representations in language regions. This approach remains largely exploratory: language cortex representations are more abstract and pose greater challenges for supervised labeling. Khanna et al. recorded single-neuron activities using Neuropixels probes in the posterior middle frontal gyrus during intraoperative mapping, and decoded sublexical structure, such as phonemic composition and syllabic structure [68]. Extending this work, Camille et al. placed electrodes on the left language regions of 14 epilepsy patients, clustering words into ten semantic categories using word embeddings and decoding category membership, with a mean decoding accuracy of 20.9% ± 3.4% [69].

Natural speech requires not only accurate transmission of semantic content, but also appropriate emotional expression and prosodic modulation. Qian et al. developed language neuroprostheses capable of restoring Chinese language and speech intonation from ECoG signals. The system uses complete tonal syllables as the basic decoding units and employs a dual-stream LSTM network to separately decode syllables and tones, with a 3-gram language model to optimize the output speech. In experiments, the system achieved 71.2% median decoding accuracy for 394 Mandarin syllables. while in real-time sentence reading tasks, the character accuracy rate reached 73.1%, with a communication rate of 49.7 characters per minute (Fig. 4d) [65]. Furthermore, humans naturally adjust their speech based on auditory feedback, necessitating ultra-low latency in decoding systems. Wairagkar et al. proposed a brain-to-voice neuroprosthetic system achieving speech synthesis with only 10 ms latency, effectively establishing the auditory-speech feedback loop [70].

### Memory neuroprostheses

Memory neuroprostheses are designed to restore or enhance memory function in individuals with impairments resulting from injury or disease. Short-term memory is formed within the hippocampus through the rapid encoding of incoming information into transient neural activity patterns. The CA3 subregion in the hippocampus supports associative encoding and pattern completion via its recurrent circuitry, whereas CA1 integrates inputs from CA3 and the entorhinal cortex and delivers memory-related signals to downstream areas. With repeated reactivation and synaptic strengthening, these transient activity patterns can be gradually transformed into long-term memory through hippocampal–cortical interactions. Accordingly, memory neuroprostheses aim to replace impaired nodes within memory circuits by recording upstream neural activities and delivering targeted stimulation to downstream neural pathways, using the electronic system to bypass the damaged signal pathway. However, given that the mechanisms underlying human memory remain incompletely understood, current memory neuroprosthetic approaches are still largely exploratory.

**Fig. 5:**
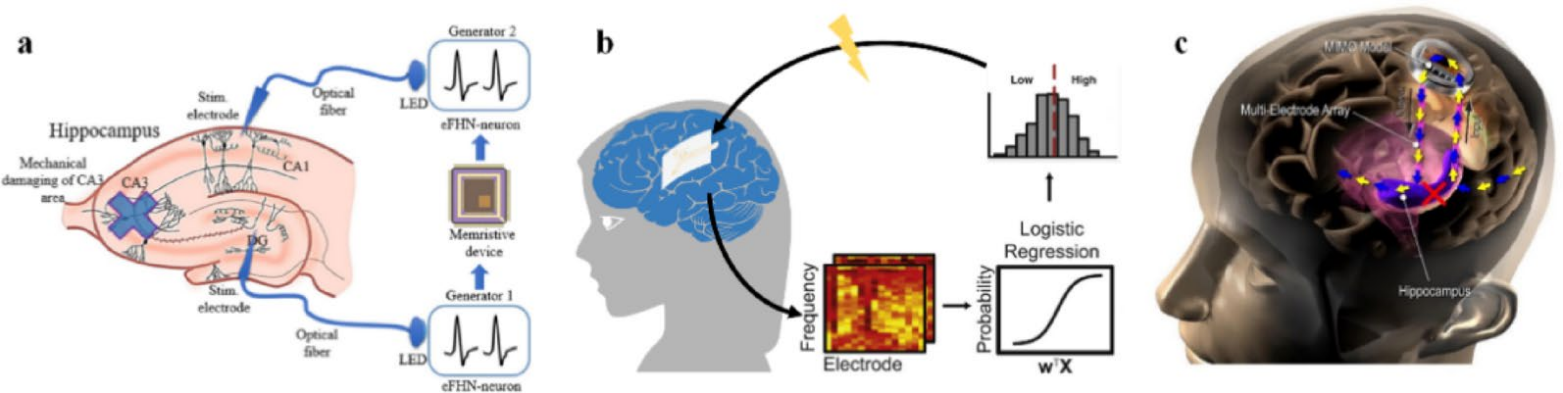
Memory neuroprostheses. **a** Memristor-based adaptive stimulation of rat hippocampal slice circuits to evoke CA1 field potential responses, reprint with permission from Ref [71]. **b** Closed-loop enhancement of memory capacity through decoding brain memory states, reprint with permission from Ref [72]. **c** MIMO model-based prediction of CA1 spiking patterns from CA3 activity for precise stimulation and improved memory performance in human subjects, reprint with permission from Ref [73]

Gerasimova et al. developed a hybrid neuromorphic system composed of two FitzHugh–Nagumo neuron oscillators coupled via an Au/ZrO₂(Y)/TiN/Ti memristive nano-device. By leveraging the memristor’s stochastic response to FHN input signals, the system can generate adaptive stimulation and successfully evoke field potential responses in the CA1 region of rat hippocampal slices (Fig. 5a) [71].

Unlike in vitro approaches, Ezzyat et al. achieved closed-loop memory improvement using real-time brain state feedback. They utilized an invasive system to modulate the brain’s “encoding states” in real-time, which reflect memory efficiency. When electrical stimulation was applied to individually determined memory-related brain regions (such as lateral temporal cortex) during low-efficiency encoding states, participants’ subsequent recall probability increased. This state-dependent effect was statistically significant at the group level. These findings demonstrate that closed-loop systems can better activate the brain to achieve memory function remodeling (Fig. 5b) [72].

Furthermore, Hampson et al. achieved a targeted invasive memory prosthesis bridging hippocampal subregions. They provided human proof‑of‑concept for a memory prosthesis by using a nonlinear multi‑input multi‑output (MIMO) model to predict CA1 firing patterns from ongoing CA3 activity. In a delayed match-to-sample task, researchers stimulated CA1 using predicted firing patterns while subjects attempted to memorize. That approach improved short-term working memory performance by 37% and long-term retention by 35% (Fig. 5c) [74].

### Olfactory neuroprostheses

Olfactory neuroprostheses aim to restore odor perception in individuals with olfactory injury. In natural olfaction, volatile odorants bind to receptors in the olfactory epithelium. The signals are then transmitted through the olfactory nerve to the olfactory bulb, where patterns of activity are formed. These patterns are further processed in higher brain areas to enable odor recognition, emotional responses, and odor-related memory. Loss of smell affects not only food enjoyment and the detection of environmental hazards, such as smoke, gas leakage, or spoiled food, but also emotion of human. Olfactory neuroprostheses capture external chemical information with artificial sensors and convert it into stimulation patterns delivered to the olfactory mucosa, olfactory bulb, or higher olfactory regions [75, 76].

The olfactory mucosa is the most peripheral and accessible target. Early studies showed that electrical stimulation of the olfactory mucosa could evoke olfactory-related neural activities, but clear subjective odor percepts were not reliably induced, because this approach depends heavily on the integrity of residual peripheral olfactory structures [77]. The olfactory bulb is considered as a more promising target, because it is located downstream in the olfactory pathway compared to the olfactory mucosa. Holbrook et al. delivered stimulation to olfactory bulb through a transethmoid pathway. This stimulation evoked odor-like percepts, such as onion-like, antiseptic-like, sour, fruity, or unpleasant, in three of five participants [78]. More recently, Mignot et al. reported a direct olfactory bulb stimulation during awake patients, and evoked a lemon-like percept, further supporting the olfactory bulb as a candidate stimulation site [79]. Higher olfactory cortices can be stimulated to produce more complex odors. Bérard et al. stimulated the medial orbitofrontal cortex in eight patients with temporal lobe epilepsy and evoked pleasant olfactory percepts, such as lemon or coffee. In three of these patients, increasing the stimulation amplitude changed odor identification, suggesting that higher cortical stimulation may modulate different odor rather than simply alter odor intensity [80]. In addition, Beyond the olfactory pathway, trigeminal nerve offers a more engineering alternative. Brooks et al. developed the stereo-smell system, which acquires signals from external gas sensors and stimulate the trigeminal nerve at the nasal septum. By modulating width and polarity of current pulse, the system encoded odor intensity and left–right directional information. In a virtual odor-source localization task, participants achieved an average localization error of 0.631 m without prior training, compared with 1.20 m under the real-odor condition [81].

Overall, the olfactory mucosa is accessible but depends on residual peripheral function. The olfactory bulb may provide more feasible method, but it requires more precise spatial and temporal stimulation. Higher olfactory cortices can encode more abstract and individualized smell, but they make stimulation harder to control. Current stimulation strategies cannot reliably evoke multiple, stable odor percepts. As a result, a deeper understanding of olfactory neural coding and more precise stimulation are essential for future progress in olfactory neuroprostheses.

### Challenges

Despite rapid advances, neuroprostheses remain far from widespread clinical adoption, with performance still inferior to natural biological function. The major challenges underlying this gap fall into three categories: mechanistic understanding, encoding/decoding strategies, and hardware systems. At the mechanistic level, functions such as motor control, visual perception, somatosensory integration, language production, and memory formation all depend on coordinated activity across multiple brain regions, yet the underlying neural mechanisms of information processing, transmission, and integration remain poorly understood. Consequently, existing neuroprosthetic systems remain largely at the stage of empirical design, making it difficult to achieve truly mechanism-driven precision engineering and optimization.

Regarding encoding and decoding strategies, current approaches lack both precision and generalizability. For the stimulation approaches, most systems rely on simple parameter modulation, which cannot effectively replicate the multi-feature coupling and spatiotemporal coordination inherent in natural neural activity. This limits the fidelity of complex sensory reconstructions, particularly for vision and touch. For the decoding approaches, existing algorithms show limited adaptability to individual differences, physiological fluctuations, and long-term signal drift, resulting in poor generalizability and frequent recalibration that severely constrains clinical utility. Furthermore, current methods cannot adequately represent advanced semantics, abstract concepts, or complex cognitive states, and struggle to achieve accurate, stable decoding with open vocabularies, large-scale information, or complex cognitive contexts.

Most critically, current hardware performance falls short of the requirements for complex neural function reconstruction. First, sensors remain inadequate in multi-modal information acquisition, spatiotemporal resolution, and chronic biocompatibility, limiting high-fidelity neural signal acquisition. Second, neural electrode throughput remains insufficient to support the large-scale, multi-regional, high-bandwidth information exchange required for complex functions. This limitation is particularly pronounced in visual, language, and memory prostheses that depend on coordination across broadly distributed networks. Furthermore, electrode stimulation precision remains restricted, as electrical stimulation is susceptible to current spread and volume conduction, inducing unintended activation of surrounding neurons and resulting in blurred or diffuse percepts. This limits high-resolution sensory reconstruction. Finally, current neuroprostheses lack robust bidirectional interaction capability. Efficient coupling remains elusive across the closed-loop pipeline of high-quality recording, real-time processing, precise stimulation, and dynamic feedback, hindering natural, stable, and intelligent human-machine integration.

## Architecture of neural prosthetic system

The survey of recent neuroprostheses revealed both promising potential and notable limitations. To bridge this gap, these fragmented challenges must be addressed through a unified engineering framework. A generalized neuroprosthetic system architecture is discussed in this section. Although motor, visual, tactile, speech, memory, and olfactory interfaces target distinct neural pathways, they collectively serve to re-establish or compensate for disrupted communication between the nervous system and the external environment. Functional divergence among these neuroprostheses can be categorized by the directionality of information flow. Devices oriented toward efferent pathways, such as motor and speech prostheses, typically translate neural activity into executable external commands or functional output. Conversely, afferent-oriented systems, such as visual, tactile, and olfactory, generally convert environmental stimuli into structured neural stimulation. To accommodate these across-layer interactions, signal processing can be coherently framed into three foundational stages: signal acquisition, signal decoding/encoding, and output execution.

**Fig. 6:**
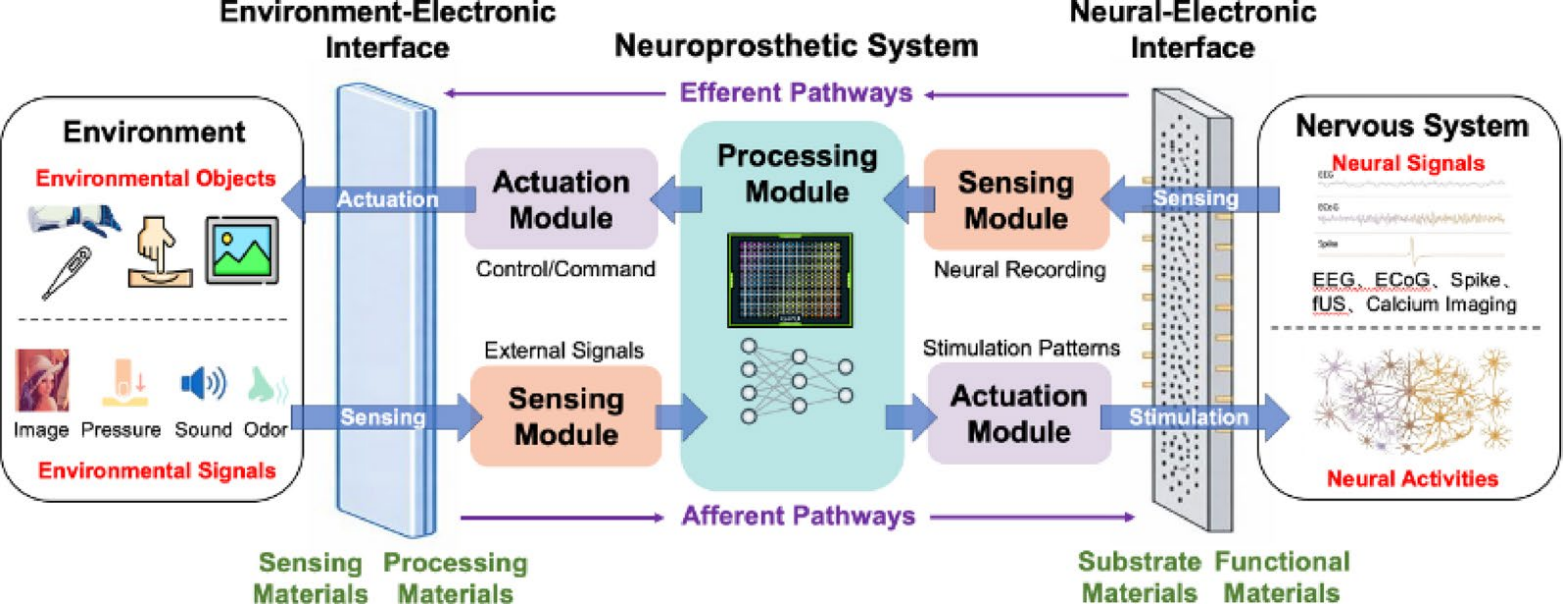
Schematic of the neuroprosthetic system

Therefore, neuroprostheses can be fundamentally conceptualized as an electronic system between the external environment and the nervous system. Efficient communication across these domains are enabled by three coordinated functional modules: (1) a sensing module for data acquisition; (2) a processing module for computing, encoding, and decoding; (3) an actuation module for output execution. Additionally, two critical interfaces underpin this architecture: the neural-electronic interface and the environment-electronic interface, as shown in Fig. 6. The external environment represents the physical world where humans acquire sensory information (e.g., light, sound, force) and serves as the target of prosthetic interaction (e.g., robotic arms). The nervous system comprises the central nervous system (brain and spinal cord) and the peripheral nervous system, which perceives and processes sensory information and generate commands. The electronic system functions as an intermediary between the nervous system and the external environment, responsible for extensive processing that decodes complex neuronal spikes into control commands (along the efferent pathway), and encodes environmental signals into neural stimulation patterns (along the afferent pathways).

### Sensing module

The sensing module is used for collecting information from the external environment or nervous system, which resides at both interfaces. At the environment-electronic interface, it serves as artificial sensory organ that replace damaged receptors, extracting information from surroundings, including light, force, sound, and temperature, and translating it into electrical signals for downstream processing. At the neural-electronic interface, the module records neural signals through sensors such as neural electrodes.

Neural electrodes are the most representative sensing module for neuroprostheses, which can be classified by their invasiveness. Non-invasive approaches such as electroencephalography (EEG) measure postsynaptic potentials from synchronized neuronal populations at the scalp. They are safe and easy to deploy, but skull-induced attenuation and spatial blurring limit resolution and reduce signal-to-noise ratios [82]. Semi-invasive electrodes such as electrocorticography, positioned in between the skull and neural tissue, exhibit higher signal-to-noise-ratio, broader bandwidth up to several hundred hertz, and improved spatial precision [83]. Invasive electrodes are implanted into the brain, enabling millisecond temporal resolution and recording both local field potentials (LFP) and action potentials with single neuron resolution [84].

Beyond electrophysiology, other sensing modalities, including magnetic, ultrasonic and optical techniques have also been explored. Magnetic approaches comprise magnetoencephalography (MEG) and functional magnetic resonance imaging (fMRI). MEG detects extremely weak magnetic fields using superconducting or optically pumped sensors, allowing non-invasive measurements with relatively high spatial fidelity. Meanwhile, fMRI infers neural activity from blood-oxygen-level-dependent (BOLD) signals and serves as the principal method for high-resolution whole-brain mapping. For instance, the spontaneous recording of alpha rhythms was reported using a cesium atomic gradiometer without magnetic shielding, achieving SNRs of 5 [85]. Additionally, typing intention decoding was achieved using MEG signals with a 32% character error rate [86]. In addition to magnetic approaches, functional ultrasound (FUS) can detect cerebral blood volume changes induced by neural activity, enabling non-invasive access to deep brain structures. For instance, Liu et al. used FUS in non-human primates to decode motion (translation vs. rotation) with up to 93% single-trial accuracy, respectively [87]. Optical approaches include functional Near-Infrared Spectroscopy (fNIRS) and Calcium Imaging are also effective and non-invasive recording technologies. fNIRS measures cerebral blood flow changes via differential absorption of near-infrared light by oxygenated and deoxygenated hemoglobin, while calcium imaging employs calcium-sensitive fluorescent indicators to transduce neural activity induced calcium dynamics into optical signals. For instance, Si et al. used fNIRS to classify positive, neutral, and negative emotional states [88]. Krishna et al. used two-photon imaging data obtained from the motor cortex of rhesus monkeys to decode the direction of arm movements online [89].

### Processing module

The processing module is responsible for decoding high-dimensional neural signals into control commands and encoding sensor information into neural stimulation. The function of this module relies on the coordinated operation of hardware and algorithms.

At the hardware aspect, neuroprosthetic information processing mainly relies on general-purpose central processing units (CPUs) and graphics processing units (GPUs). At the algorithmic aspect, traditional decoding methods, such as template matching, linear discriminant analysis (LDA), and Wiener filtering, have been widely applied to extract intentions from neural signals. Model-based approaches construct neural response models based on electrophysiological properties, whereas adaptive strategies aim to maintain stable perceptual outcomes despite dynamic changes in neural states or electrode conditions. In recent years, deep-learning approaches such as convolutional neural networks (CNNs) and recurrent neural networks (RNNs) have shown superior performance in capturing the spatiotemporal structure of high-dimensional neural signals. Overall, processing modules form the computational core linking sensing and actuation, and provide critical support for the operation of neuroprostheses.

These hardware units offer mature solutions for system design and algorithm development, but generally lack optimization for efficiency and form factor. As a result, a separate packaging such as a backpack is often required to carry around the processing module by the patients, compromising portability and convenience for daily use. A critical bottleneck in conventional CPU/GPU-based platforms stems from the inherent separation of memory and processing units in von Neumann architectures, where frequent data transfer incurs substantial energy consumption and processing latency. To address these limitations, emerging material-enabled computing paradigms such as in-memory computing and neuromorphic computing have attracted growing attention. These platforms can significantly reduce the computing overhead and accelerate the processing speed with highly parallel matrix operations. Moreover, since these computations occur in analog circuits, they allow direct interfacing between analog sensing and actuation modules, thus eliminating the costly analog-to-digital (ADC) and digital-to-analog conversions (DAC) in these embedded systems. Thereby, they provide improved energy efficiency, faster response times, and seamless integration with biological systems.

### Actuation module

The actuation module forms the terminal stage of information flow in neuroprostheses and serves two main roles: (1) converting decoded neural intentions into actions through efferent pathways, and (2) delivering encoded sensory information back to the nervous system through afferent pathways. Along efferent pathways, actuators replace the damaged functions of patients. such as robotic arms, exoskeletons and speech synthesizers. Along afferent pathways, actuators represented by electrodes typically delivere electrical stimulation to the nervous system. By adjusting stimulation parameters, such as frequency, amplitude, and pulse width, external information can be encoded and delivered into neural system. However, electrical stimulation often activates mixed neuronal populations around the electrode, which makes cell-type-specific modulation difficult. To enhance stimulation precision, alternative approaches are actively being explored, such as optogenetics. Optogenetic methods employ genetic engineering to express photo-sensitive proteins in selected neurons, achieving precise control of neural activity via light.

Additionally, non-invasive stimulation methods are also being investigated. Focused ultrasound uses the mechanical or thermal effects to modulate neural activity, offering non-invasive access with deep penetration. For example, a non-invasive ultrasound retinal prosthesis using a 16 × 16 phased array transducer was reported, demonstrating the potential to restore vision without surgical procedure [90]. Meanwhile, transcranial magnetic stimulation (TMS) has been used clinically for deep brain modulation. For example, Kim et al. developed magnetoelectric nanodiscs combining magnetostrictive and piezoelectric materials, which enabled wireless and targeted neuromodulation of deep brain regions in mice after injection of small nanoparticle doses [91].

In summary, the actuation modules serve as the terminal stage of neuroprostheses and are critically influenced by both sensing and processing modules. Future development is directed toward enhanced precision and reduced invasiveness, particularly in neural stimulation strategies.

## Interface materials for neuroprostheses

From the perspective of biological information flow, the nervous system senses external information and sends it to the brain for perception, while the brain sends commands to control the body. Different types of interactions dictate the design of the electronic devices. For instance, different neural signals, such as spike (action potential), local field potential (LFP) and EEG reflect neural activities from micro-scales to macro-scales. Each of these signals exhibits diverse characteristics that require unique design considerations. For spike detection, invasive electrodes with functional surface coatings should be used to improve charge injection capacity for better signal-to-noise ratio. Meanwhile, conductive gels are commonly used for EEG recording to establish low impedance electrical contact with the scalp but normally require post-recording cleanup. In contrast, dry electrodes with specialized materials or structural designs are being developed to improve user convenience. Therefore, the overall performance of the neuroprosthetic system depends not only on sensing, processing and actuation modules mentioned above, but more importantly on the key interfaces where the electronic devices are carefully designed to align with the requirements of biological systems. The performance of these interfaces directly determines the efficiency, precision, and long-term stability of signal acquisition, transmission, and feedback stimulation. Overall, neuroprosthetic systems comprise two critical interfaces: (1) the neural-electronic interface, and (2) the environment-electronic interface (Fig. 6). The neural–electronic interface is mainly represented by neural electrodes, while the environment–electronic interface is mainly represented by various sensors.

### Substrate materials for electronic-neural interface

**Fig. 7:**
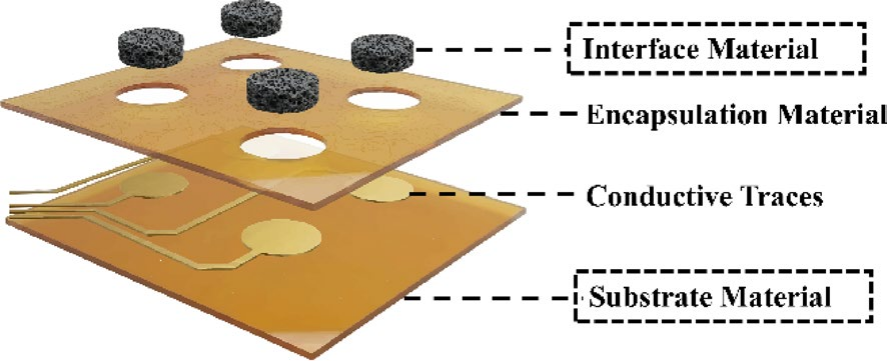
Schematic diagram of typical structure of neural electrodes

The neural–electronic interface, represented by neural electrodes, has two key components: the electrode substrate and the electrode interface (Fig. 7). The electrode substrate primarily provides mechanical support, spatial positioning and integration. Key requirements include sufficient mechanical strength and process compatibility, chemical stability in the corrosive environment, and good biocompatibility for long-term implantation [92].

Early substrate materials primarily focused on mechanical support and implantation convenience. Metal microwires, typically made of tungsten and platinum-iridium alloys, became classic tools in electrophysiology research because their high axial stiffness enables direct insertion into deep brain regions without supporting shuttles. However, the fabrication of microwires often relies on manual or semi-automated methods, leading to limited precision and poor consistency, which makes it difficult to achieve high-density channel integration within confined spaces. In addition, microwire arrays are prone to uncontrollable displacement during implantation due to a lack of rigid constraint, resulting in insufficient targeting precision. More critically, microwires fail to meet biocompatibility standards for chronic implantation because of the immense mechanical mismatch between probes and brain tissue. Consequently, chronic physiological micromotion induces continuous mechanical cutting, resulting in glial encapsulation and signal attenuation [93].

**Fig. 8:**
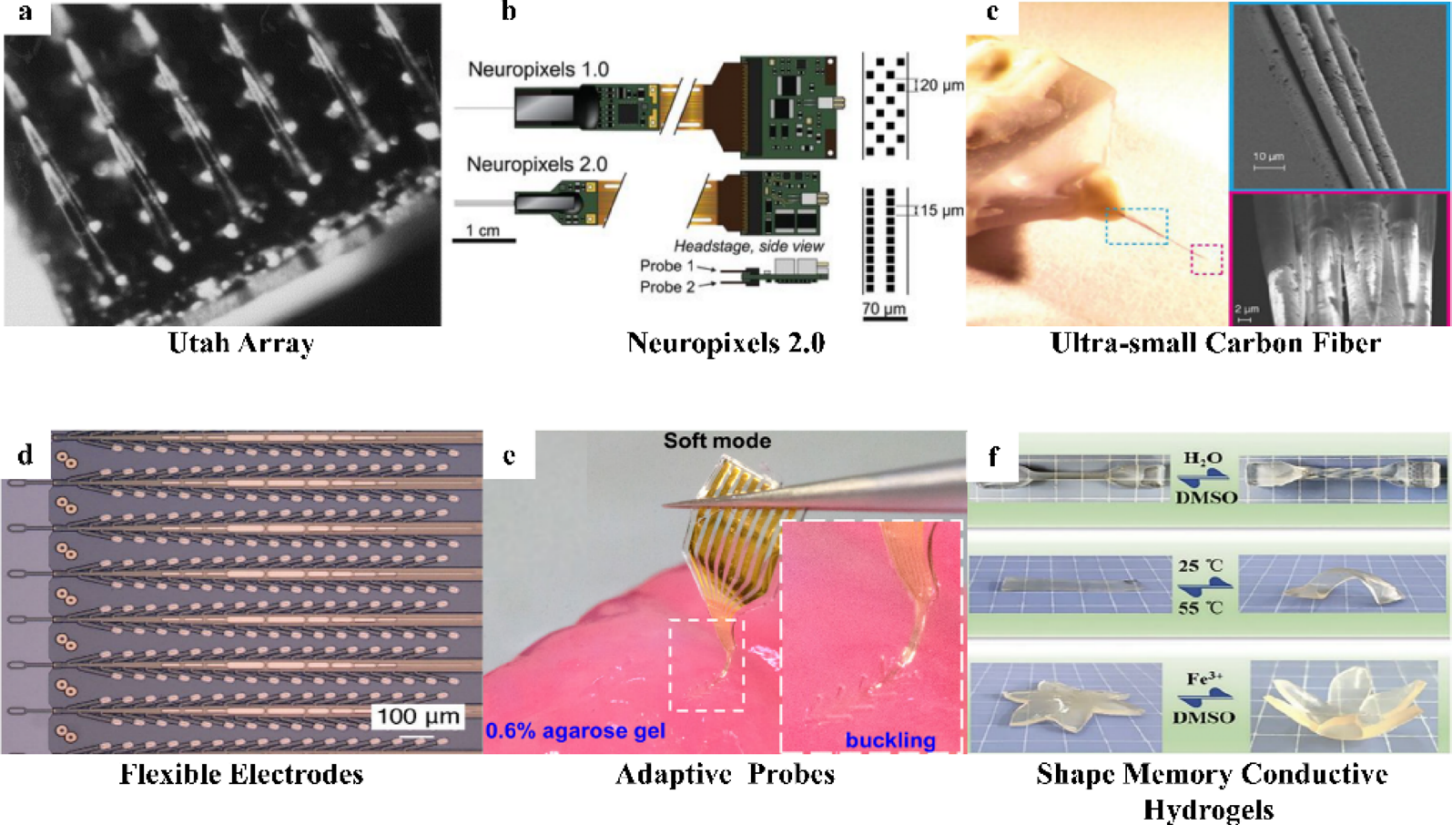
Overview of substrate materials for neural electrodes. **a** Silicon-based electrode: Photograph of a Utah Array [95]. **b** Silicon-based electrode: Photograph of a Neuropixels 2.0 [96]. **c** Carbon fiber electrode: Photograph of an ultra-small carbon fiber electrode, reprint with permission from Ref [100]. **d** Flexible electrode: Flexible electrode: Neuralink probes, reprint with permission from Ref [101]. **e** Adaptive probe: A mechanically adaptive and deployable intracortical probe, reprint with permission from Ref [102]. **f** Hydrogel-based devices: A shape memory conductive hydrogel by constructing Fe3 + interactions in PVA-catechol hydrogel matrix, reprint with permission from Ref [103]

To overcome the limitations of microwire electrodes in precision and channel throughput, silicon-based microelectrode arrays based on Micro-Electro-Mechanical Systems (MEMS) technology were developed. Representative platforms, including the Michigan electrode and Utah arrays, use MEMS processes to achieve high-throughput integration and submicron fabrication precision within compact structures, enabling long-term stable recording in brain (Fig. 8a) [94, 95]. For instance, the Neuropixels 2.0 probe integrates 5,120 recording sites across four shanks (Fig. 8b) [96], allowing large-scale tracking of neural activity in rodent brains. Silicon-based electrodes have also demonstrated strong potential in clinical applications. For example, Feng et al. successfully implanted Utah arrays in elderly patients, helping them control VR systems and exoskeletons via MI for rehabilitation training [97]. Subsequently, the same team further decoded complex Chinese character writing trajectories from motor cortical activity [98], demonstrating reliable information recording of Utah arrays. Similarly, rigid silicon probes are prone to damaging brain tissue in the micro-motion environment due to their mechanical mismatch with neural tissue, potentially triggering severe chronic inflammatory responses that limit their service life in chronic implantable brain–computer interfaces (BCIs) [99].

To address the mechanical mismatch between rigid electrodes and soft brain tissue, ultra-micro carbon fibers were proposed to achieve effective mechanical compliance through geometric miniaturization, which represented a shift toward to flexible electrode. Guitchounts et al. fabricated electrodes using carbon fibers with diameters < 5 μm, whose extremely small cross-section greatly lowers bending stiffness. These fibers enable electrode to “float” with brain tissue micromotion, and long-term experiments showed that these electrodes could record single-neuron signals stably for months with minimal glial scarring (Fig. 8c) [100]. However, carbon fiber electrodes remain difficult to produce at scale because their fabrication relies on assembling individual fibers which fail to meet the demands for thousands of channels.

To achieve both mechanical flexibility and high-density integration, flexible electrodes based on polymer films were developed, representing a major transformation in electrode morphology. Materials such as polyimide (PI), parylene and polydimethylsiloxane (PDMS) possess low mechanical modulus, which contributes to improved biocompatibility, and are fully compatible with MEMS processes that enable low-cost production of high-density electrode arrays [104]. For instance, Song et al. showed that ultra-thin flexible devices can conform closely to cortical surfaces, significantly reducing immune responses while enhancing signal quality [105]. A major step toward clinical translation came from Musk et al., a PI-based high-density flexible probe called Neuralink to be implanted accurately by precision surgical robots (Fig. 8d) [101]. However, implantation method relying on shuttle needles can still cause brain injury. To address this, Wang et al. developed a “mechanically adaptive” probe based on shape-memory polymers, which exhibit a unique thermosensitive phase-change mechanism. This probe maintains rigid during implantation and transforms into soft after insertion at body temperature, reducing stiffness by orders of magnitude (Fig. 8e) [102]. This rigid-to-flexible transition provides both surgical stability and long-term tissue compatibility.

In recent years, substrate materials have increasingly evolved toward ultra-thin, ultra-flexible designs to improve biocompatibility and enable higher levels of functional integration. In terms of ultra-thin and ultra-flexible, Guo et al. leveraged a single-layer 500-nm ultrathin PI film as the electrode substrate, yielding an overall device thickness of ~ 1 μm. The probe exhibited a bending stiffness of ~ 2.6 × 10^-^¹⁴N·m², comparable to that of axons, maintaining reliable local field potential and single-unit recordings in mouse cortex for at least 30 days [106]. Separately, Sheng et al. employed perfluoropolyether dimethacrylate, a high-performance synthetic elastomer, to construct tissue-level-soft, submicrometre-thick mesh microelectrode arrays, achieving ultra-compliant mechanics with a maximum stress of ~ 68 kPa under ~ 29% tensile strain. The electrode enabled continuous 5-day in vivo electrophysiological recording in the embryonic axolotl brain with isolatable single-neuron spiking activity [107]. In terms of multi-modal integration, 2D materials such as graphene provide a new material platform for combining electrical and optical neural interfaces because of their exceptional flexibility and high optical transparency. Kuzum et al. exploited the highlight transmittance of graphene to develop a transparent flexible electrode array to simultaneously record electrophysiological signals and optical imaging of neuronal calcium activity [108]. In addition, bio-based materials such as silk fibroin and hydrogels are increasingly used to construct long-term stable electrode substrates due to their natural biocompatibility and tunable mechanical properties. Kim et al. employed silk fibroin films as temporary support layers for ultrathin electrode. After implantation, controlled degradation of fibroin generates capillary forces that drove the electrodes to shrink-wrap the cortical surface [109]. This mechanism enabled tight adhesion to complex brain gyri, eliminated interfacial gaps and achieved highly conformal contact with high SNR that is difficult to reach with conventional electrodes. Hydrogel-based devices offer another route toward tissue-like interfaces. Liu et al. showed that hydrogels can be engineered with Young’s moduli closely matching that of brain tissue, giving the implants near “mechanical invisibility” [110]. Yuk and Fu further demonstrated that the porous mesh structure of hydrogels provides high permeability, allowing free diffusion of glucose, oxygen and growth factors, and thereby avoiding nutrient blocking in surrounding neural tissue (Fig. 8f) [103, 111]. The comparison of representative substrate materials above is shown in Table 1.

**Table 1:** Comparison of representative substrate materials

Type	Materials	Advantages and Limitations	References
Metallic materials	Tungsten; Platinum–iridium alloys	High electrical conductivity and good stability; poor MEMS compatibility; limited chronic biocompatibility	[93]
Silicon-based materials	Single-crystal silicon; Highly doped silicon	High MEMS compatibility; limited biocompatibility due to mechanical mismatch	[94–97, 99]
Carbon fiber materials	Carbon fibers	High electrical conductivity and stability; biocompatibility; poor MEMS compatibility	[100]
Polymer materials	Polyimide; Parylene; Polydimethylsiloxane	Flexible materials with biocompatibility; difficult implantation	[101, 104, 105]
	Shape-memory polymers	Rigid during implantation and soft in vivo; easy implantation and biocompatibility; difficult fabrication	[102]
	Perfluoropolyether dimethacrylate	Ultrathin and ultrasoft; biocompatibility; difficult implantation and fabrication	[107]
Bio-based materials	Silk fibroin	Assists flexible electrodes during implantation; dissolves in the brain after implantation	[109]
	Hydrogels	High biocompatiblity; difficult implantation and limited chronic stability	[103, 110, 111]

### Functional materials for electronic-neural interface

Interfacial functional materials at electrode surfaces mediate electron-ion exchange and are engineered to enhance the fidelity of communication between electronic systems and neural tissue. Key research priorities include optimizing electrochemical properties, such as high charge-injection capacity and low interfacial impedance, while concurrently ensuring chemical stability to prevent coating degradation and biocompatibility to avoid neurotoxic effects [112].

**Fig. 9:**
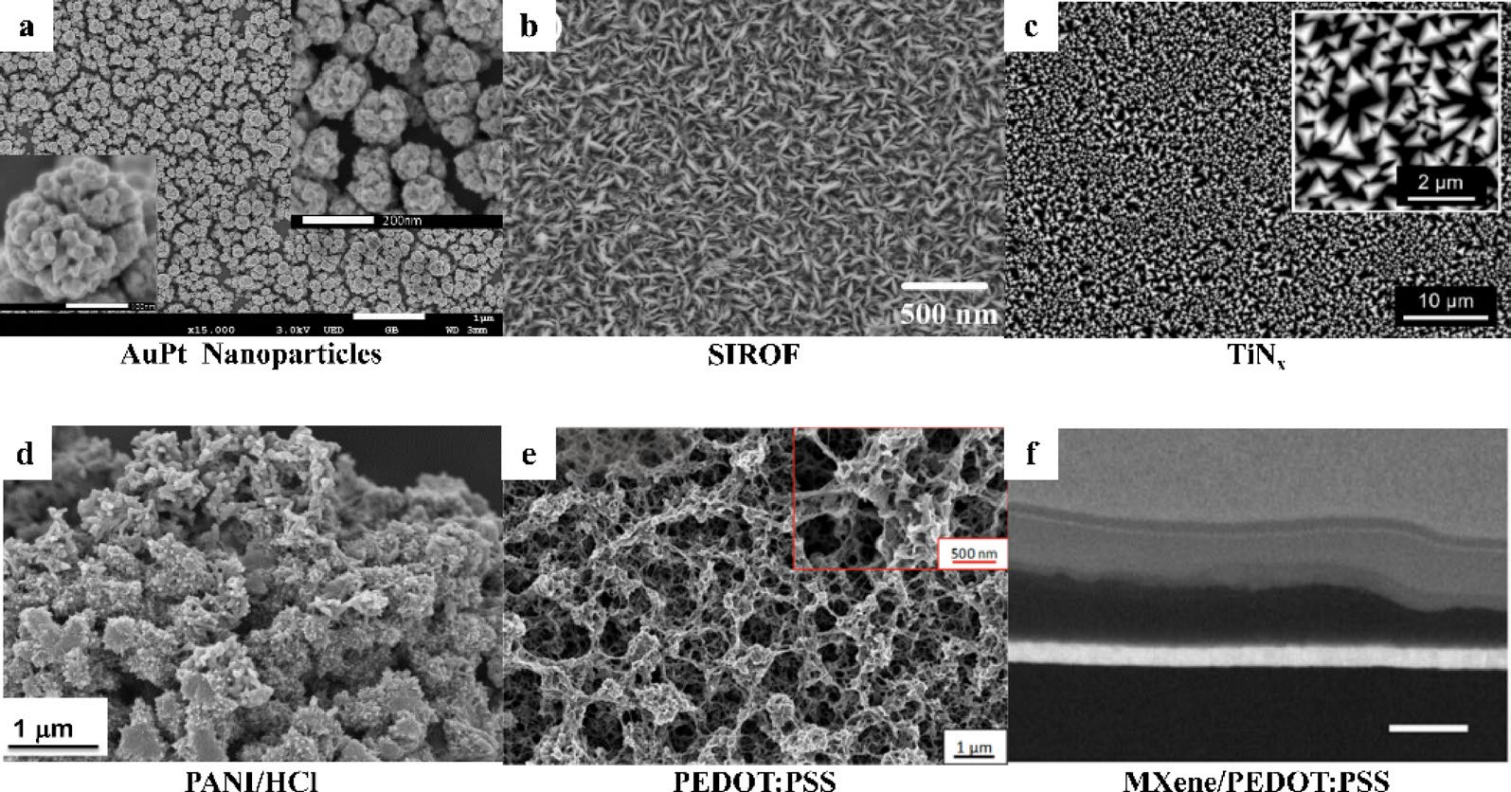
Overview of interface materials for neural electrodes. **a** SEM image of AuPt nanoparticles, reprint with permission from Ref [117]. **b** SEM image of Sputtered Iridium Oxide Film (SIROF), reprint with permission from Ref [118]. **c** SEM images of the ultra-porous TiN deposited onto Ti6Al4V, reprint with permission from Ref [121]. **d** SEM images of PANI/HCl, reprint with permission from Ref [122]. **e** SEM image of PEDOT: PSS polymer coating showing its characteristic porous nanostructure, reprint with permission from Ref [123]. **f** SEM micrograph of MXene/PEDOT: PSS fibers, reprint with permission from Ref [124]

The interface functional materials initially relied mainly on metals, such as platinum and iridium, which also served as the substrate materials. However, as electrode dimensions continuously shrink, the effective surface area of bare metal decreases sharply, leading to a rapid increase in interfacial impedance that fails to meet the requirements of neural signals detection. This challenge has driven the development of advanced interface functional materials which enhance electron–ion coupling, reducing interfacial polarization and increasing charge transport capacity [113, 114]. Traditional metal interfaces mainly optimize electrical performance through surface roughening. A classic example is platinum black, which forms a nanoporous structure by electrodeposition to increase effective surface area and lower impedance [115]. However, platinum black often suffers from poor mechanical adhesion which make it prone to delamination, and potential tissue toxicity. To address these limitations, more mechanically stable nanostructured metals have been developed. Brüggemann et al. developed a pure gold nanostructured microelectrode with vertically aligned nanopillar arrays using a template-assisted electrodeposition method. The large effective surface area of the nanopillar structure significantly reduced interfacial impedance and doubled the amplitude of recorded neural signals compared with planar gold electrodes, demonstrating that microstructured design alone can markedly improve recording stability. Furthermore, long-term cell experiments showed that these electrodes maintained both structural integrity and stable electrical performance [116]. Similarly, Zhao et al. developed a gold–platinum (AuPt) alloy coating with a nanoporous structure through an electro-co-deposition process. This alloy interface showed better mechanical stability than platinum black, and lowered background thermal noise from 34.1 µV to 7.5 µV, substantially enhancing the ability of microelectrodes to detect weak neural signals (Fig. 9a) [117].

Meanwhile, to meet the demand of high-current density stimulation, metal compound materials are widely utilized as interfacial materials due to their efficient and stable charge transfer capability. For example, iridium oxide (IrO_x_) provides a large Faraday Capacitance through the reversible redox reaction between Ir³⁺ and Ir⁴⁺, enabling a charge injection capacity exceeding 3 mC/cm² (Fig. 9b) [118]. In contrast, Titanium nitride (TiN) provides stable double-layer capacitance called non-Faraday Capacitance owing to its columnar porous structure. Previous studies have shown that TiN-coated electrodes exhibit an impedance nearly 1/10 that of smooth metals and remain resistant to corrosion after millions of stimulation pulses, thereby achieving a balance between electrical performance and long-term physicochemical stability (Fig. 9c) [119–121].

Conductive polymers (CPs) extend the electrical performance of neural electrodes through their mixed electron–ion transport mechanisms and generally exhibit higher biocompatibility than metals or metal oxides. CPs allow ions to move throughout their three-dimensional molecular network, enabling efficient charge injection via bulk redox reactions. Harris et al. demonstrated that poly(3,4-ethylenedioxythiophene) (PEDOT) coatings can reduce electrode impedance by 30 to 50 times and substantially improve SNR in chronic recordings [113]. Despite these advantages, many CPs, including polypyrrole (PPy) and polyaniline (PANI) (Fig. 9d) [122], undergo pronounced volumetric changes during charge–discharge cycles due to ion insertion and extraction, resulting in mechanical stress and film delamination [125]. Although the electrochemical stability of poly(3,4-ethylenedioxythiophene) : poly(styrenesulfonate) (PEDOT: PSS) has been significantly improved (Fig. 9e) [123, 126], cracking and delamination remain challenging under long-term, high-intensity stimulation. To address these challenges, Oçafrain et al. introduced functionalized self-assembled monolayers (SAMs) as molecular bridges. One end of the SAM forms a stable covalent bond with the metal substrate, such as an Au-S linkage, while the other end, for example an EDOT functional group, participates in polymer chain growth, chemically anchoring the conductive polymer film to the electrode surface and enhancing mechanical robustness [127]. In a complementary approach, Wustoni et al. incorporated two-dimensional transition metal carbides, known as MXenes, as macromolecular co-dopants during electropolymerization. The negatively charged surface terminations of MXenes provide stable charge compensation for PEDOT holes, effectively confining polymer chains, suppressing volumetric expansion, and mitigating long-term degradation and delamination (Fig. 9f) [124].

As the demands for high electrical performance, mechanical compatibility, biocompatibility and long-term stability increasingly exceed the capabilities of single-component materials, composite systems have emerged as a major focus. Conductive composite material systems, represented by carbon nanomaterial–conducting polymer composites, construct “nanotunnels” by embedding carbon nanomaterials into CP matrices. This approach can significantly improve charge transport efficiency and mechanical stability of single polymers under long-term high-intensity electrical stimulation [128]. Lv et al. employed PtNPs/PEDOT: PSS/MWCNTs composite-modified microelectrodes, reducing impedance from 438 kΩ to 5.7 kΩ at 1 kHz, increasing charge storage capacity to 31.5 mC/cm² (over 100-fold higher than bare gold electrodes), and achieving a dopamine detection sensitivity of 1.13 µA/µM/cm². Following implantation into the hippocampal CA1 region of APP/PS1 mice, the device enabled stable, long-term simultaneous 40 Hz electrical stimulation and concurrent recording of dopamine dynamics and neural activity [129]. Furthermore, He et al. systematically compared the doping effects of single-walled carbon nanotubes (SWCNTs) and multi-walled carbon nanotubes (MWCNTs) in PEDOT: PSS, and confirmed that SWCNTs have a larger electrochemical active area and more active sites, thus outperforming MWCNTs [130].

High biocompatibility remains a critical consideration for next-generation interface functional materials. Cuttaz et al. demonstrated that polypropylene dioxythiophene (ProDOT) incorporating bioactive peptides as dopants can maintain low impedance while simultaneously endowing the interface with anti-inflammatory and neurotrophic activity [131]. In parallel, Ziai et al. emphasized the importance of intelligent responsiveness for achieving biocompatibility, as such materials can dynamically adapt to microenvironmental disturbances following implantation, marking a shift from neural interfaces with passive compatibility toward active interaction with living tissue [132].

Finally, the growing demand for wireless neuromodulation has stimulated exploration of ferroelectric composite interfaces, exemplified by P(VDF–TrFE)/BaTiO₃. These systems leverage the piezoelectric effect to convert incident ultrasound into local electrical stimulation to suffice for activating neuronal calcium channels, offering a potential pathway toward passive, wireless next-generation neural interfaces [120]. The comparison of representative functional materials above is shown in Table 2.

**Table 2:** Comparison of representative functional materials

Type	Materials	Advantages and limitations	References
Nanostructured metals	Platinum black	High electrical conductivity; poor mechanical stability and poor adhesion	[115]
	Gold nanopillar arrays		[116]
	Gold–platinum alloy nanoparticles		[117]
Metal compounds	Iridium oxide	Excellent stimulation performance; difficult fabrication	[118]
	Titanium nitride		[119–121]
Conductive polymers	PEDOT; PPy; PANI	High biocompatibility and conductivity; limited stability, especially for electrical stimulation	[113, 122, 123, 125, 126]
	Functionalized self-assembled monolayers SAMs		[127]
	MXenes		[124]
Composite materials	Nanostructured metals/metal compounds/conductive polymers	Integrated advantages of multiple material systems; difficult fabrication	[128, 129]
Biological materials	Bioactive peptides	High conductivity; anti-inflammatory	[131]
Intelligent materials	Intelligent responsive materials	Dynamically adapt to microenvironmental disturbances; shift from passive compatibility to active interaction	[132]

### Sensing materials for environment-electronic interface

At the environment-electronic interface, materials research is increasingly directed toward the transduction and processing of external information. These efforts aim to enhance the sensitivity, resolution and multi-modal perception capabilities of external information acquisition, while simultaneously ensuring high biocompatibility with human tissues.

**Fig. 10:**
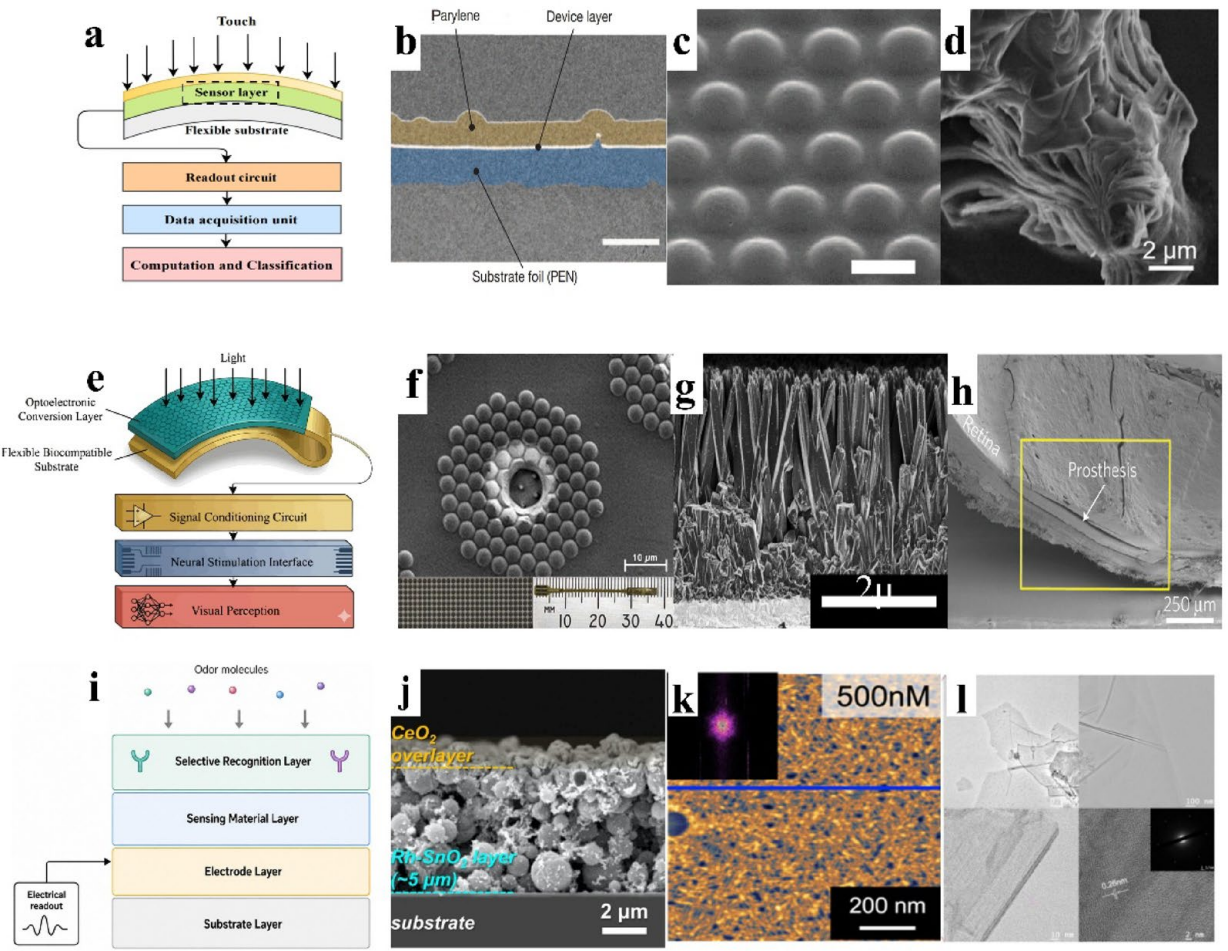
Tactile, visual and olfactory systems and sensing materials. **a** Cross-sectional schematic of the artificial tactile system, illustrating the sensor layer, readout circuit, and flexible substrate assembly, reprint with permission from Ref [133]. **b** SEM of an ultrathin transistor encapsulated with 800-nm parylene diX-SR near the neutral strain plane (scale bar: 2 μm), reprint with permission from Ref [137]. **c** Tilted SEM of e-skin with interlocked microdome arrays (10 μm diameter, 4 μm height, 12 μm pitch; scale bar: 10 μm), reprint with permission from Ref [141]. **d** SEM of the TaS₂/Cu₂S heterojunction nanostructure (scale bar: 2 μm), reprint with permission from Ref [142]. **e** Schematic of the artificial vision system. **f** SEM of retinal prosthesis electrodes, showing high-density tiles with 85 SiNWs capped with IrOx per electrode, reprint with permission from Ref [143]. **g** Side-view SEM of Au-TiO₂ nanowire arrays, reprint with permission from Ref [144]. **h** Cross-sectional SEM of prosthesis embedded in the subretinal space after 6 months (scale bar: 50 μm), reprint with permission from Ref [145]. **i** Schematic of the artificial olfactory system. **j** SEM image of the CeO₂/Rh–SnO₂ film, reprint with permission from Ref [146]. **k** Atomic force microscopy (AFM) images of self-assembled GR3R peptide structures on a graphite surface, reprint with permission from Ref [147]. **l** Photograph of MXene/peptide-modified Au electrodes, reprint with permission from Ref [148]

Sensing materials of neuroprostheses are mainly developed for tactile, visual and olfactory prosthetic applications (Fig. 10). An ideal tactile interface seeks to reproduce the extraordinary sensing capability of biological skin. Beyond high responsiveness to mechanical deformation, materials must resolve dynamic textures, subtle slip and thermal cues [133]. The early tactile sensors, largely based on rigid silicon MEMS platforms, lacked the mechanical compliance required to conform to complex surfaces [134, 135]. The transition from rigid to flexible electronics therefore marked a foundational step toward intimate biointegration and accurate perception. Pioneering this direction, Someya et al. employed pentacene organic field-effect transistors to realize a large-area flexible pressure-sensing matrix, establishing an early prototype of electronic skin. Yet the achievable bending radius remained limited to millimeter scales (~ 4 mm) [136]. Through geometric engineering, Kaltenbrunner et al. subsequently produced an ultrathin device only 2 μm thick on a 1.2 μm polyethylene naphthalate substrate, enabling bending radius down to 5 μm and allowing the sensor to laminate onto fine epidermal microstructures such as fingerprints (Fig. 10b) [137]. In addition, to address the large strain fracture and electrical signal distortion caused by joint movement, Xu et al. introduced the conjugated-polymer/elastomer phase-separation induced elasticity strategy. By nanoconfining semiconducting polymers within soft SEBS elastomers, the approach suppresses crystallization-induced brittleness and preserves stable electrical performance even under 100% strain [138].

As mechanical compliance improved, research focus shifted from simple pressure readout toward biomimetic architectures capable of richer force interpretation. Xi et al. constructed a three-layer PDMS protrusion–rubber–electrode array using piezoresistive rubber which enable decoupling of three-dimensional forces, and identify slip through high-frequency signal analysis [139]. Boutry et al. further advanced this concept with carbon-nanotube/polyurethane pyramidal electrodes that emulate the dermal-epidermal interface, achieving rapid and sensitive discrimination between normal and shear stresses [140]. To capture even subtler features such as fine texture, Park et al. drew inspiration from fingerprint ridges and developed a ferroelectric e-skin with interlocked microstructures. Surface roughness is translated into amplified vibration signatures, allowing reliable spectral differentiation between materials such as glass and sandpaper. Simultaneously, the pyroelectric response of poly(vinylidene fluoride-trifluoroethylene) (P(VDF-TrFE)) enables co-localized measurement of pressure and temperature, approximating the integrated sensory functionality of human skin (Fig. 10c) [141]. Together, these strategies illustrate how structural biomimicry enhances the dimensionality and fidelity of tactile encoding.

Beyond sensitivity and functionality, practical deployment demands autonomy and portability. Addressing this, Wang et al. proposed a triboelectric self-powered scheme based on PDMS and fluorinated ethylene propylene. Mechanical contact and separation directly generate usable electrical output, eliminating the need for external power [149]. Building upon the concept of energy independence, Yang et al. achieved self-powered humidity sensing by using a TaS₂/Cu₂S-based triboelectric nanogenerator (TENG): mechanical contact–separation between a polyethylene terephthalate (PET) layer and TaS₂/Cu₂S electrodes generates electrical output to drive the sensor. With the TaS₂/Cu₂S nanoflower heterostructure, the device delivered high humidity sensitivity (S = 3.08 × 10⁴, 11–97% RH) and a fast response (~ 2 s), enabling practical demonstrations such as environmental humidity and respiration monitoring (Fig. 10d) [142].

The development of visual prosthetic materials is directed toward the construction of artificial retinal interfaces that simultaneously deliver high photoelectric conversion efficiency, favorable biocompatibility and dense pixel integration. Mathieson et al. pioneered a photovoltaic strategy based on silicon photodiode arrays. In a 70 μm pixel unit, the incorporation of iridium oxide stimulation electrodes enabled a charge injection density of up to 1.5 mC/cm², allowing reliable high-density activation under near-infrared illumination well within ocular safety limits. Nevertheless, the relatively weak optical absorption of planar silicon constrains the number of photocarriers that can be generated, thereby limiting further improvements in injection efficiency [150].

To address this bottleneck, subsequent efforts turned to micro- and nano-structure of the light-absorbing layer. Bosse et al. introduced vertically aligned silicon nanowire arrays, exploiting their strong light-trapping capability. Multiple scattering events and waveguide modes markedly prolong the residence time of photons inside the material, leading to more than a threefold enhancement in photoconversion and charge injection, and correspondingly reducing the illumination threshold required to elicit neural responses (Fig. 10f) [143]. Building on this principle, Tang et al. advanced a biomimetic design inspired by the geometry of retinal rod and cone cells. Gold-decorated TiO₂ nanowires prepared via a hydrothermal route form vertically ordered structures. These vertically aligned nanowires construct a “one-dimensional optical waveguide” structure similar to a biological photoreceptor at the microscale, and combined with the surface plasmon resonance effect of gold nanoparticles, significantly improving quantum efficiency and charge separation capability (Fig. 10g) [144]. While these studies focused primarily on maximizing optoelectronic performance, the mechanical incompatibility between rigid inorganic materials and soft neural tissue remains a persistent concern. To alleviate retinal damage induced by modulus mismatch, Maya-Vetencourt et al. employed the conjugated polymer poly(3-hexylthiophene) (P3HT) to build a flexible artificial retina. The intrinsic compliance of the polymer allows intimate conformal contact with the curved ocular surface, thereby mitigating post-implantation inflammatory reactions. Functionally, the device operates through a photocapacitive mechanism: photogenerated carriers accumulate at the interface to form an electric double layer, which modulates neuronal membrane potential via capacitive coupling. By circumventing faradaic charge transfer associated with conventional metal electrodes, this approach reduces electrochemical corrosion and enhances the long-term safety and temporal responsiveness of stimulation (Fig. 10h) [145].

Beyond improvements in efficiency and biophysical compatibility, recent work has sought to harness the intrinsic physicochemical dynamics of materials to endow prostheses with local information-processing capability, thereby easing the demands on backend computation. Luo et al. combined an ionogel with PPy nanoparticles to construct a heterojunction device. Upon illumination, the resulting temperature gradient drives Li^+^ and TFSI^−^ to migrate at different rates, generating a built-in electric field and an ionic photocurrent. The photocurrent response can be dynamically tuned by the intensity, number and temporal spacing of optical pulses, allowing synapse-like plasticity to be emulated at the device level. This supports spatiotemporal integration and processing at the sensor edge, such as motion-trajectory tracking and speed estimation, advancing visual prosthetics from passive perception towards integrated sensing, memory and computing [151]. Parallel advances are pushing visual prostheses toward self-powered operation and expanded perceptual modalities. Wang et al. developed a retinal neuroprosthesis based on tellurium nanowire networks, where defect-induced internal asymmetry—arising from features such as Sn substitution and Te vacancies—creates a built-in driving force for carrier separation. This architecture generates substantial photocurrent even at zero external bias, enabling autonomous functionality. Owing to tellurium’s narrow bandgap (~ 0.3 eV), the detectable spectrum extends beyond visible wavelengths into the near-infrared II window (~ 1550 nm). In vivo experiments confirmed that the implant not only restored daylight vision in blind mice but also conferred sensitivity to infrared radiation, effectively broadening the natural limits of mammalian sight [31].

Olfactory sensing materials are designed to detect gas molecules with high sensitivity and selectivity. Seiyama et al. propose the metal-oxide semiconductor (MOS) for gas detection [152], and the miniaturized MOS-based sensor arrays have been widely used because of small size, low power consumption, and improved selectivity (Fig. 10j) [146]. In parallel, conductive polymer/carbon material arrays have been developed for the detection of volatile organic compounds, which highlights the importance of conductive composites in olfactory sensing [153, 154].

More recently, biological elements, such as peptides, have been embedded into olfactory sensors to improve molecular selectivity. Homma et al. integrated olfactory receptor motif-derived peptides with graphene field-effect transistors for odor detection. In this design, the peptides provided specific molecular recognition, while graphene enabled sensitive electrical signal transduction (Fig. 10k) [147]. Similarly, MXene/peptide sensor arrays combine two-dimensional materials which have the large surface area and high conductivity, with the peptides. This method improved the sensitivity and selectivity of volatile organic compounds detection (Fig. 10l) [148]. Artificial olfaction has also been extended toward integrated sensing and memory. Yin et al. developed an artificial olfactory memory material based on the conductive metal–organic framework Ce-HHTP (cerium-2,3,6,7,10,11-hexahydroxytriphenylene). In this system, continuous channels and Ce active sites were used to bind oxygen-containing guest molecules in different ways, leading to short-term memory for alcohols and selective long-term memory for aldehydes [155]. In the future, the olfactory sensing materials are moving toward higher sensitivity and selectivity for the detection of complex gas samples. The comparison of representative sensing materials above is shown in Table 3.

**Table 3:** Comparison of representative sensing materials

Application	Name	Features/advantages and disadvantages	References
Tactile	Silicon-based materials	High sensitivity and easy fabrication, but poor conformability to the skin surface	[134, 135]
	Organic flexible materials	Flexible and conformable to the skin surface	[136, 137]
	Three-dimensional elastic materials	Capable of detecting three-dimensional forces and recognizing multiple tactile perceptions	[138, 139]
	Nanomaterials	Sensitive and rapid response; capable of detecting subtle tactile stimuli	[140, 141, 149]
Visual	Silicon-based optoelectronic materials	Reliable and stable	[150]
	Nanomaterials	High photoelectric conversion efficiency	[143, 144]
	Polymeric materials	Flexible conformability and biocompatibility	[145, 151]
Olfactory	Metal oxide semiconductors	Miniaturization, low cost, and good stability	[146, 152]
	Conductive polymer composites	Sensitive to volatile organic compounds	[153, 154]
	Biopeptides	Ultra-high selectivity	[147, 148]
	Conductive metal–organic frameworks	Capable of recognition and memory, with great potential for intelligent olfaction	[155]

### Processing materials for environment-electronic interface

The human brain employs a hierarchical architecture of neural processing layers to extract meaningful information from abundant sensory inputs. Consequently, signals in specific cortical areas are highly abstract and nonlinear, differing drastically from raw sensory input. As a result, extensive signal processing is required to encode sensor inputs into delicate stimulation patterns. The advancement of artificial intelligence offers unique toolsets for producing high quality stimulation signals. However, these algorithms often demand high computing power from bulky computer servers, making them impractical for daily clinical use. One prominent challenge arises from the physically separated memory and processing unit design in the von Neumann architecture. Frequent data transfers between these units lead to high transmission latency and excessive energy consumption, making existing computing systems extremely inefficient for edge applications. To overcome this bottleneck, Compute-in-Memory (CIM) architecture has emerged in recent years as a highly efficient architecture for AI applications. By enabling matrix operations directly within the memory arrays, CIM significantly reduces transmission overhead and enables fast matrix operations that substantially accelerates neural network processing.

**Fig. 11:**
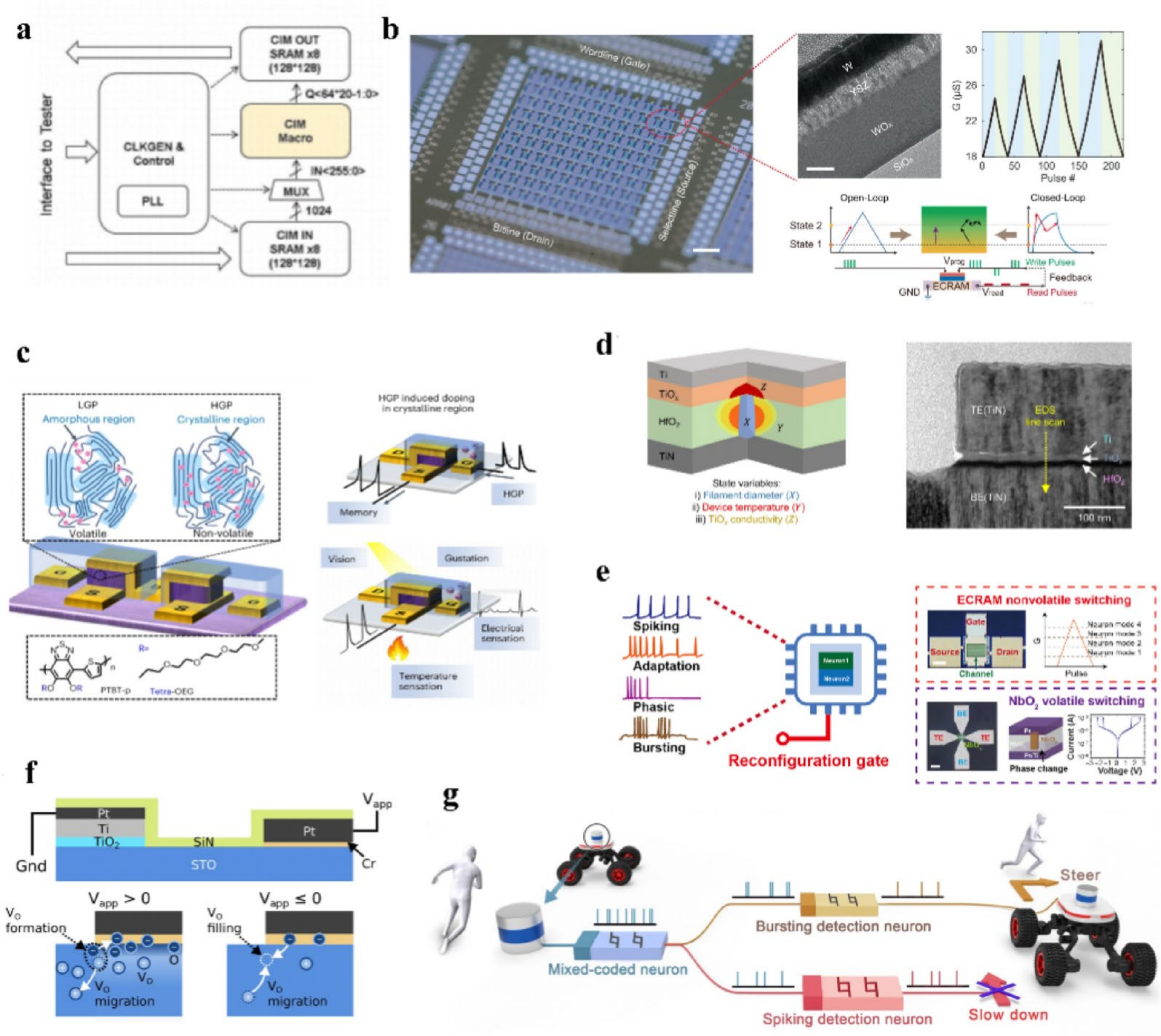
Processing materials and application. **a** The architecture of compute-in-memory chip based on SRAM, reprint with permission from Ref [156]. **b** An open-loop electrochemical memory array, reprint with permission from Ref [159]. **c** Device structure and function of volatile/non-volatile organic electrochemical transistors, reprint with permission from Ref [160]. **d** Structure of a third-order memristor, reprint with permission from Ref [164]. **e** The chip of supporting various spiking modes (left) and the core components, including a nonvolatile ECRAM memory device and a volatile NbO2 device (right), reprint with permission from Ref [165]. **f** Memristor Structure and Switching Mechanism, reprint with permission from Ref [166]. **g** Control the steering and actuating of the robot based on memristors, reprint with permission from Ref [167]

Among various CIM architectures, digital static random-access memory (SRAM) based CIM represents a short-term and high-reward approach that leverages mature CMOS foundry process to build high performance chip with potential for mass production. For instance, Chih et al. reported a fully digital SRAM-CIM macro in a 22 nm process, achieving an energy efficiency of 89 TOPS/W (Fig. 11a) [156]. However, the large physical footprint of SRAM cells imposes significant constraints on its on-chip scalability to match growing software needs. Emerging non-volatile memory devices, such as memristors, offer novel and highly scalable alternatives. Compared with SRAM, these devices have demonstrated superior advantages in scalability, data retention and multi-level storage, making them suitable to host large neural networks on-chip. For instance, Liu et al. developed an analog memristor-based CIM chip and demonstrated fully parallel multiply-accumulate (MAC) operations [157]. Meanwhile, to address the non-ideal characteristics of analog devices such as conductance drift and write noise, Yao et al. employed a hybrid training strategy to effectively compensate for accumulated errors. They achieved over 96% recognition accuracy in a fully hardware-implemented memristor convolutional neural network, approaching software accuracy [158]. To enhance the programming capability suitable for real-time adjustment, Chen et al. developed an electrochemical memory devices (ECRAM) which demonstrated highly accurate open-loop programming capability in arrays (Fig. 11b) [159], offering unique capability for on-chip learning.

In addition to CIM, additional functionalities of emerging devices have been explored to further enhance the processing efficiency in low-power scenarios such as neuroprosthetic systems. For example, in-sensor computing was proposed to explore CIM devices with sensing capability. For instance, Wang et al. developed organic electrochemical transistors (OECTs) featuring crystalline-amorphous ion channels (Fig. 11c) [160]. These devices switch between a volatile receptor mode (responsible for multi-modal sensing of ions and light) and a non-volatile synapse mode (providing 10-bit analog storage). Cui et al. proposed a ferroelectric photo-sensing network (FE-PS-NET), leveraging the tunable optical response of ferroelectric sensors to synchronize image acquisition and processing at the sensing end [161]. Huang et al. monolithically integrated optoelectronic memristor arrays with Si CMOS to construct a 128 × 8 fully integrated array, accomplishing optical sensing, weight storage, and computation on a single hardware substrate [162].

By harvesting the bio-plausible features of these devices, more natural interaction with the biological systems could also be achieved for efficient neuroprosthetic system design. For instance, Chen et al. combined pressure-activated organic electrochemical transistors with artificial mechanoreceptors to create low-voltage artificial organic afferent nerve, which was applied in closed-loop tactile feedback [163]. Similarly, Park et al. introduced an additional TiOₓ resistive switching layer into HfO₂ memristors to fabricate devices exhibiting third-order switching complexity (Fig. 11d) [164]. This enabled artificial sensory systems to demonstrate habituation and sensitization at the device level, allowing for selective responses to significant stimuli without relying on external processors.

In the meantime, using these devices for neuromorphic computing has been widely adopted. By adopting novel material systems that more closely mimic biological properties (e.g., memristors, phase-change materials, and organic semiconductors). These materials can directly simulate the electrochemical behaviors of biological neurons and synapses at the device level [168]. For instance, Xiao et al. constructed reconfigurable spiking neurons using NbO₂ threshold-type memristors and ECRAMs (Fig. 11e) [165]. By coupling fast-slow dynamics, they achieved multiple firing modes—including phasic, adaptive, and bursting—within a single compact circuit. Similarly, Li et al. tightly integrated nonlinear electrochemical elements with solid-state memristors to create ion-electron hybrid artificial neurons [169]. This design expanded the adjustable pulse frequency range by five orders of magnitude and enabled direct sensing of fluid flow, temperature, and chemical composition. Meanwhile, Weilenmann et al. utilized SrTiO₂ memristors operating in the non-filamentary, low-conductance regime to simultaneously exhibit multiple long-term and short-term synaptic mechanisms within a single device (Fig. 11f) [166]. Furthermore, Yang et al. employed memristor-based Hodgkin–Huxley neuron circuits in sensing-motor control loops, moving tonic spiking and bursting phenomena from mere device demonstrations to active participation in robot obstacle avoidance for input encoding and control decision-making (Fig. 11g) [167].

Despite these potentials, emerging processing materials still face several critical barriers for practical neuroprosthetic use. A primary challenge lies in their intrinsic non-idealities, including conductance drift, noise, nonlinear or asymmetric analog-state updates, all of which create mismatches between hardware and software algorithms, and compromise computational precision and reliability. In addition, many material systems still suffer from limited long-term stability and endurance under repeated programming or prolonged operation. Another challenge is that the behavior of these materials is often highly sensitive to microscopic defects and interfacial variations due to lack of mature and reliable fabrication processes, leading to inconsistent electrical characteristics and reduced controllability during operation. Future progress will therefore depend on improving material uniformity, stabilizing interfaces, and achieving more precise control over coupled ionic/electronic processes, which together may enable more reliable scaling of these devices into large-scale integrated processing hardware for practical use in neuroprosthetic systems.

## Conclusions

Neuroprostheses hold great potential to revolutionize neurological therapies and enhance human capabilities. With innovations in advanced materials, electronics and artificial intelligence, the field is poised for further breakthroughs. Currently, decoding-based systems, such as motor and language prostheses, have displayed faster progress than stimulation-based sensory prostheses such as touch and vision, while most of neuroprostheses have yet to achieve seamless integration with the biological systems. These limitations highlight persistent challenges that restrict the broader development and clinical deployment of neuroprostheses.

Although artificial sensors detect isolated features with high accuracy, they still lag behind biological receptors in several aspects. First, it is still difficult to build artificial sensors with high spatial resolution to emulate retinal or skin sensations, resulting in coarse visual and tactile reconstructions. Second, current prosthetic sensors lack information fusion capabilities, resulting in fragmented perceptions of the rich stimuli. Third, biological sensors perform sophisticated front-end feature extraction, whereas artificial sensors typically lack profound pre-processing capabilities, imposing substantial computational burdens on downstream processing units and compromising system’s real-time performance.

Meanwhile, the interaction between neural and electronic systems is hindered by significant mismatches. First, low electrode count and limited spatial resolution cause severe information undersampling, restricting decoding of complex functions. Second, chronic implantation triggers glial scarring, progressively degrading recording fidelity and stimulation efficacy. Third, modal divergence remains a critical obstacle that causes substantial information loss and precludes the faithful replication of natural neural coding.

From a system perspective, the advancement of neuroprostheses is a multi-dimensional engineering task that spans from neural mechanisms and materials to devices and systems. A mechanistic understanding of neural coding, circuit dynamics, and plasticity provides the necessary foundation. In decoding, insights into neural mechanisms will shift BCIs from data-driven classification toward mechanistically explainable decoding, enabling high resolution spatiotemporal modeling of cross-regional dynamics. In encoding, understanding neuronal coding strategies including spike timing-dependent codes and transient responses will facilitate the design of bio-plausible neuromorphic stimulations, facilitating more naturalistic encoding.

Materials and devices form the physical foundation of high-throughput, chronically stable neuroprostheses. Sensors are evolving toward high-sensitivity, multimodal integration and enhanced biocompatibility, and sensor architectures are shifting toward integrated sensory-computing paradigms, enabling efficient front-end operations. Simultaneously, high-density, biocompatible neural electrodes are transitioning from rigid to flexible materials that match the mechanical properties of soft neural tissue, minimizing microtrauma and enhancing chronic stability. At the electrode interface, engineering nanomaterials are designed that combine high stability, biocompatibility, and superior electrochemical performance, enabling high signal-to-noise ratio recording and efficient neural stimulation.

At the system level, the realization of closed-loop interaction is paramount for advanced prosthetic functionality, where efficient edge computing is indispensable to this objective. Through in-memory computing architectures or neuromorphic chips, signal processing can be executed directly at the implant site, drastically reducing latency and power consumption. Moreover, establishing sensory-motor bidirectional loops is essential for achieving naturalistic control. By dynamically adjusting stimulation parameters based on real-time neural feedback, these systems can significantly enhance modulation precision, providing profound brain-machine integration and mutual adaptation.

Overcoming these core challenges will enable efficient, stable, and naturalistic communication between electronic systems and the nervous system for advanced neuroprostheses. It is evident that a systematic perspective is critical, requiring synergistic efforts from mechanistic insights, innovations in materials, and engineering of high-performance closed-loop systems. Through breakthroughs in these critical domains, future neuroprostheses will evolve beyond mere functional substitution to become a hybrid bio-electric intelligent system that seamlessly integrate together.

## Data Availability

Not applicable.

## Abbreviations

ECoG: Electrocorticography
LFP: Local field potential
EEG: Electroencephalography
SNR: Signal-to-noise ratio
MEG: Magnetoencephalography
fMRI: Functional magnetic resonance imaging
FUS: Functional ultrasound
fNIRS: Functional near-infrared spectroscopy
CPU: Central processing unit
ASIC: Application-specific integrated circuit
GPU: Graphics processing unit
DRAM: Dynamic random access memory
CIM: Compute-in-memory
SRAM: Static random-access memory
MAC: Multiply-accumulate
ANN: Artificial neural network
SNN: Spiking neural network
LIF: Leaky integrate-and-fire
TMS: Transcranial magnetic stimulation
MEMS: Micro-electro-mechanical systems
BCI: Brain–computer interface
PI: Polyimide
PDMS: Polydimethylsiloxane
PEDOT: Poly(3,4-ethylenedioxythiophene)
PEDOT:PSS: Poly(3,4-ethylenedioxythiophene): polystyrene sulfonate
PPy: Polypyrrole
PANI: Polyaniline
SAM: Self-assembled monolayer
SWCNT: Single-walled carbon nanotube
MWCNT: Multi-walled carbon nanotube
ProDOT: Poly(propylene dioxythiophene)
P(VDF-TrFE): Poly(vinylidene fluoride-trifluoroethylene)
TENG: Triboelectric nanogenerator
PET: Polyethylene terephthalate
P3HT: Poly(3-hexylthiophene)
ME: Motor execution
MI: Motor imagery
RP: Retinitis pigmentosa
AMD: Age-related macular degeneration
FDA: Food and Drug Administration
IRIS: Intelligent retinal implant system
cpd: Cycles per degree
BVT: Bionic vision technology
UEA: Utah electrode array
V1: Primary visual cortex
V4: Visual area V4
JND: Just noticeable difference
SEEG: Stereo-electroencephalography
PNS: Peripheral nervous system
USEA: Utah slanted electrode array
SA1: Slowly adapting type I
RA: Rapidly adapting
PC: Pacinian corpuscles
TIME: Transversal intrafascicular multichannel electrode
ICMS: Intracortical microstimulation
SSVEP: Steady-state visually evoked potential
ERP: Event-related potential
WER: Word error rate
AUC: Area under the curve
CAR: Character accuracy rate
MIMO: Multi-input multi-output
